# Circulating Biomarkers for Predicting Cardiovascular Disease Risk; a Systematic Review and Comprehensive Overview of Meta-Analyses

**DOI:** 10.1371/journal.pone.0062080

**Published:** 2013-04-22

**Authors:** Thijs C. van Holten, Leonie F. Waanders, Philip G. de Groot, Joost Vissers, Imo E. Hoefer, Gerard Pasterkamp, Menno W. J. Prins, Mark Roest

**Affiliations:** 1 Laboratory for Clinical Chemistry and Hematology, UMC Utrecht, Utrecht, The Netherlands; 2 Philips Research Laboratories, Philips, Eindhoven, The Netherlands; 3 Research and Development, Future Diagnostics, Wijchen, The Netherlands; 4 Experimental Cardiology Department, UMC Utrecht, Utrecht, The Netherlands; Universidad Peruana de Ciencias Aplicadas (UPC), Peru

## Abstract

**Background:**

Cardiovascular disease is one of the major causes of death worldwide.

Assessing the risk for cardiovascular disease is an important aspect in clinical decision making and setting a therapeutic strategy, and the use of serological biomarkers may improve this. Despite an overwhelming number of studies and meta-analyses on biomarkers and cardiovascular disease, there are no comprehensive studies comparing the relevance of each biomarker. We performed a systematic review of meta-analyses on levels of serological biomarkers for atherothrombosis to compare the relevance of the most commonly studied biomarkers.

**Methods and Findings:**

Medline and Embase were screened on search terms that were related to “arterial ischemic events” and “meta-analyses”. The meta-analyses were sorted by patient groups without pre-existing cardiovascular disease, with cardiovascular disease and heterogeneous groups concerning general populations, groups with and without cardiovascular disease, or miscellaneous. These were subsequently sorted by end-point for cardiovascular disease or stroke and summarized in tables. We have identified 85 relevant full text articles, with 214 meta-analyses. Markers for primary cardiovascular events include, from high to low result: C-reactive protein, fibrinogen, cholesterol, apolipoprotein B, the apolipoprotein A/apolipoprotein B ratio, high density lipoprotein, and vitamin D. Markers for secondary cardiovascular events include, from high to low result: cardiac troponins I and T, C-reactive protein, serum creatinine, and cystatin C. For primary stroke, fibrinogen and serum uric acid are strong risk markers. Limitations reside in that there is no acknowledged search strategy for prognostic studies or meta-analyses.

**Conclusions:**

For primary cardiovascular events, markers with strong predictive potential are mainly associated with lipids. For secondary cardiovascular events, markers are more associated with ischemia. Fibrinogen is a strong predictor for primary stroke.

## Introduction

Atherothrombosis is one of the major causes of death worldwide [1]. Upon rupture of an atherosclerotic plaque, a hemostatic response is initiated that could lead to infarction causing ischemia downstream. Assessment of cardiovascular disease risk supported by biomarker analysis is a primary requirement to stratify those at high-risk and for optimized treatment of patients.

Large cohort studies are crucial for cardiovascular disease risk estimation with the use of biomarkers, and confirmation of results in independent populations is desirable. Many results from different studies have become available over time, which makes it challenging to assess those markers that consistently keep a predictive value. Meta-analyses combine the results from different studies and present one aggregate score for a risk marker in question, but these studies have also been performed in large numbers. This systematic review presents a comprehensive overview of serological biomarkers for cardiovascular disease events and stroke in cardiovascular disease naïve populations (being primary cardiovascular events), and cardiovascular disease events and stroke in populations with a history of cardiovascular disease (being secondary cardiovascular events) investigated in meta-analyses of the past 24 years [2]. It compares the relevance of the most commonly studied biomarkers used to assess the risk for ischemic cardiovascular event and stroke. The selection of meta-analyses was restricted to prospective studies only, as pooled results from cross-sectional and retrospective case-control studies overestimate the risk for the marker in question. To our knowledge, this is the first systematic review on meta-analyses for biomarkers of atherothrombosis.

## Materials and Methods

**Figure 1:**
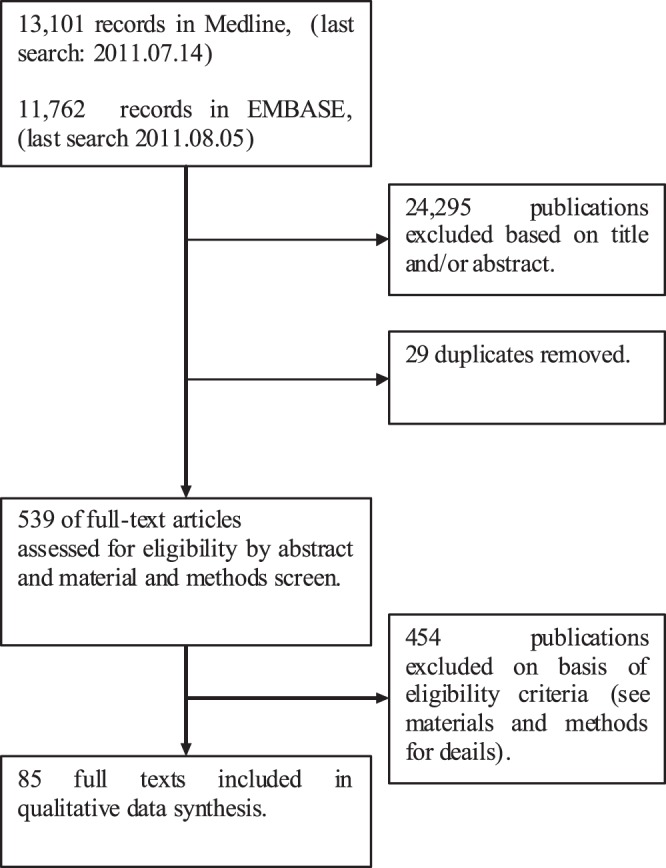
PRISMA flow diagram.

A literature search on published studies from 1988 to 2011 has been performed in Medline (using the advanced search option in Pubmed), and Embase (see flow diagram in Figure 1). A protocol for the search and data abstraction was set up and discussed with one skilled epidemiologist, and three established investigators for consensus. The search terms were related to “arterial ischemic events” and “meta-analyses”, and were set up broadly to reduce the possibility that publications that use trivial nomenclature would be missed (see Supplementary Methods in File S1). We have taken the PRISMA statements as a framework for reporting the systematic review [3].

Titles and abstracts were screened on “meta-analyses of prospective studies”, “arterial ischemic disease”, and “levels of circulating markers” (Figure 1, Step 1.). Duplicates were removed from the search results (Figure 1, Step 2.). Eligibility of the papers was assessed by reading the abstracts and material and methods section of the publications (Figure 1, Step 3.). Studies were considered as “not eligible” if they were not prospective (e.g. cross-sectional, retrospective studies), reported other than levels of circulating markers (e.g. alleles, or prediction models), investigated the risk relating to all cause death or hemorrhagic stroke, did not explicitly report the pooled results in the text, figures, or tables; did not pool data from 2 prospective studies or more, did not report risk in relation to levels of the investigated marker (e.g. comparison of marker levels in case and control group), were unpublished reports (e.g. abstracts, posters), or that were not available online via either Medline or Embase (in total 11 meta-analyses were irretrievable online).

After the first selection round, manuscripts were selected for a full text screen. The result of the meta-analysis was extracted, which was reported either by odds ratio (OR), relative risk (RR), relative risk ratio (RRR), or hazard ration (HR), together with the follow up period. In addition, the following parameters were abstracted: the type of investigated end-point(s), to what group the risk applied and how this group was defined (e.g. tertiles, cut-offs), nature of pooled studies (e.g. individual patient data (IPD), cohorts), whether there had been adjustment for other risk factors, presence of statistical heterogeneity or heterogeneity mentioned by the authors, how the pooling was performed (either by regression, Cox-regression, random effects model, fixed effects model, or inverse variance weighted combined risks), number of patients (the amount of cases within the pooled cohorts was preferred but if this was not present the total cohort size was given), number of pooled cohorts, and which population was represented in the results (a population with or without pre-existing cardiovascular disease, a specific subgroup population, or the general population).

If both unadjusted and adjusted results were reported, the adjusted results were abstracted. If risks for more quantiles were reported, only the most extreme was used. If subgroup meta-analyses were reported in one publication (e.g. different age groups or specific gender), these were abstracted unless these were excluded according to the earlier specified criteria. If the number of cases was not reported in the manuscript, calculations were performed by hand. Whenever in the meta-analysis review it was stated that heterogeneity is present between the cohorts, the heterogeneity was recorded as yes. When it was reported that heterogeneity was absent or it was not mentioned, the heterogeneity was recorded as no. If heterogeneity between cohorts was reported and both random effects and fixed effects analysis was performed, the random effects results were abstracted. Stratification of the meta-analyses was performed on population based studies, cohorts without pre-existing cardiovascular disease, cohorts with pre-existing cardiovascular disease, pooled results from populations with and without pre-existing cardiovascular disease, and specific subgroups.

The evaluation criteria for novel risk markers as described by Hlatky et al. 2009 were used as guide to set up evaluation parameters for meta-analyses [4]. We consider the following parameters indicative for the clinical value and quality of the different meta-analyses:

- There should be relevant stratification of the researched individuals. Groups with, or without previous cardiovascular disease are clinically more relevant than groups representing the general population, or meta-analyses where cohorts with and without cardiovascular disease were pooled for one result.
- The prediction is preferred to be expressed as a hazard ratio, rather than an odds ratio, relative risk, or relative risk ratio as it considers the event rate, and not the difference in number of events at one specific time point.
- Pooled end-points should not be too diverse, or at least clinically relevant. Pooling of diverse end-points complicates the interpretation of the results.
- The novel risk marker should be able to predict risk beyond the established risk markers, and therefore it should add statistical value in a model where other risk factors are included.
- The result of a meta-analysis becomes more reliable with increasing number of events and is even more convincing when a large number of cohorts are used, especially when in absence of heterogeneity between the pooled cohorts.
- If heterogeneity of the results is present, this should be addressed by conservative pooling of the results using a random effects model (see http://www.cochrane.org/). Statistical power of risk assessment depends on the number of outcome events, and therefore reporting of the number of events rather than total study size is preferred.

## Results

A total of 24.863 publications were screened, which were available online in the period June the 10^th^ of 2011 to August the 5^th^ of 2011. After a screen on title and abstract and subsequent removal of 29 duplicates, a total of 539 publications remained (Figure 1). After monitoring the abstracts and material and methods, 85 publications remained with 214 meta-analyses. On basis of cohort characteristics and end point 9 different types of meta-analyses were identified, which are summarized in Table 1, Table 2, Table 3 and Tables S1–9 in File S1. Meta-analyses for cardiovascular disease events that were performed with studies from groups without pre-existing cardiovascular disease are presented in Table 1 and Table S1 in File S1. Meta-analyses for cardiovascular disease events that were performed with studies from groups with pre-existing cardiovascular disease are presented in Table 2 and Table S2 in File S1. Meta-analyses reported for stroke events in populations without cardiovascular disease are presented in Table 3 and Table S3 in File S1. Pooled results for stroke events in populations with pre-existing cardiovascular disease are provided in Table S4 in File S1. Results from studies with heterogeneous populations being general populations, populations with and without pre-existing disease, and miscellaneous groups for either cardiovascular or stroke events are summarized in the Tables S5–9 in File S1. The tables are organized into categories of markers (e.g. markers related to hemostasis), and per category in descending order of result. The studies reporting on populations only without pre-existing cardiovascular disease, reporting on populations only with pre-existing cardiovascular disease, for either cardiovascular disease or stroke (Table 1–3, Table S1–3 in File S1) are considered most clinically relevant, and therefore are discussed in this review. Meta-analyses reporting on stroke in populations only with pre-existing cardiovascular disease are not discussed in this review, as only two meta-analyses were found in this category and are too few to draw any conclusions upon.

**Table 1:** Selection of meta-analyses of cohorts without pre-existing cardiovascular disease on markers for cardiovascular disease risk.

Marker	Risk Applies To	Risk	Results	95% ci	N Patients	N Cohorts	Reference
***Diabetes related***							
Glucose post load	Above: 7.8 mmol/L	RR	1.58	1.19–2.10	1,467 cases	7	[31]
Glycated hemoglobine (HBA(1c))	HbA1c level: 0.7	RR	1.58	1.22–2.06	1,366 cases	7	[32]
***Hemostasis***							
Fibrinogen	1 g/L increase	HR	2.33	1.91–2.84	992 cases	31	[5]
Fibrinogen	1 g/L increase	HR	1.93	1.79–2.08	7,118 cases	31	[5]
***Hormones***							
Vitamin D (serum 25-OH D)	Decrease in different predefined categories	HR	1.83	1.19–2.80	2,007 cases	5	[9]
Vitamin D (serum 25-OH D)	Decrease in different predefined categories	HR	1.54	1.22–1.95	756 cases	4	[9]
***Inflammation***							
CRP^1^	Top vs bottom tertile	RR	2.43	2.10–2.83	3,181 cases	12	[6]
CRP	Top vs bottom tertile	OR	1.58	1.48–1.68	7,068 cases	22	[33]
***Lipids***							
ApoB^2^	Top vs bottom tertile	RR	1.99	1.65–2.39	6,920 cases	19	[7]
ApoB/ApoAI ratio	Top vs bottom tertile	RR	1.86	1.55–2.22	3.730 cases	7	[7]
HDL^3^	0.33 mmol/L decrease	HR	1.83	1.65–2.03	1,198 cases	23	[8]
Triglycerides	Top vs bottom tertile	OR	1.72	1.56–1.90	10,158 cases	29	[34]
HDL	0.33 mmol/L decrease	HR	1.63	1.44–1.85	764 cases	23	[8]
ApoAI	Bottom vs top tertile	RR	1.62	1.43–1.83	6,333 cases	21	[7]
Non-HDL cholesterol	43 mg/dL increase	HR	1.59	1.36–1.85	12,785 cases	68	[35]
ApoB	29 mg/dL increase	HR	1.58	1.39–1.79	4,499 cases	22	[35]
Non-HDL cholesterol	1.53 unit increase	HR	1.50	1.38–1.62	4,499 cases	22	[35]
Non-HDL cholesterol	1 mmol/L decrease	HR	0.66	0.61–0.71	1,198 cases	23	[8]
Cholesterol/HDL	1.33 units decrease	HR	0.60	0.56–0.64	1,198 cases	23	[8]
Cholesterol	1 mmol/L decrease	HR	0.58	0.56–0.61	5,561 cases	61	[8]
Non-HDL cholesterol	1 mmol/L decrease	HR	0.57	0.52–0.62	764 cases	23	[8]
Cholesterol/HDL	1.33 units decrease	HR	0.56	0.51–0.60	764 cases	23	[8]
Cholesterol	1 mmol/L decrease	HR	0.44	0.42–0.48	1,309 cases	61	[8]

1 CRP: C-reactive protein.

2 Apo: apolipoprotein.

3 HDL: high density lipoprotein.

**Table 2:** Selection of meta-analyses of cohorts with pre-existing cardiovascular disease on markers for cardiovascular disease risk.

Marker	Risk Applies to	Risk	Results	95% ci	N Patients	N Cohorts	Reference
***Hemostasis***							
vWF^4^	Top vs bottom tertile	OR	1.6	1.0–2.5	723 cases	8	[36]
***Inflammation***							
Hs^5^-CRP	1 mg/L>hs-CRP>3 mg/L	OR	5.65	1.71–18.73	477 total	4	[13]
hs-CRP	1 mg/L>hs-CRP>3 mg/L	OR	2.76	1.38–5.55	386 total	3	[13]
CRP	Top vs bottom tertile	RR	1.97	1.78–2.17	6,485 cases	83	[10]
CRP	Top vs bottom tertile	RR	1.5	1.1–2.1	604 cases	3	[37]
***Ischemia***							
cTn^6^T+cTnI	Above: cTnT 0.1–0.2 ng/mL;cTnI 0.1–3.1 ng/mL	OR	9.39	6.46–13.67	160 cases	10	[12]
BNP^7^+NT^8^-proBNP	Above: BNP 116 gp/mL, NT-proBNP 227.5 pg/mL	OR	7.9	4.7–13.3	75 cases	5	[38]
cTnI	Above: unknown	RR	5.7	1.8–19	882 cases	4	[39]
cTnI	Above: different per study	OR	4.94	3.9–6.2	1,168 cases	13	[40]
cTnT+cTnI	Above: cTnT 0.1–0.2 ng/mL;cTnI 0.1–0.6 ng/mL	OR	4.93	3.77–6.45	1,602 cases	16	[12]
cTnT	Above: 0.1–0.2 ng/mL	OR	4.58	3.8–5.5	1,965 cases	16	[40]
cTnT	Above: 0.1–0.2 ug/L	OR	4.4	3.0–6.5	163 cases	4	[41]
cTnT	Above: 0.1–0.2 ug/L	OR	4.3	2.8–6.8	96 cases	7	[41]
cTnI	Above: 0.03 ug/L–3.1 ug/L	RR	4.2	2.7–6.4	n.a.	9	[42]
cTnT	Above: unknown	RR	3.8	2.6–5.5	1,292 cases	12	[39]
cTnT+cTnI	Above: cTnT 0.1–0.2 ng/mL; cTnI 0.1–3.1 ng/mL	OR	3.11	2.59–3.74	201 cases	21	[12]
cTnT	Above: 0.1–0.2 ng/mL	OR	2.86	2.35–3.47	1,330 cases	3	[12]
cTnT+cTnI	Above: cTnT 0.1–0.2 ng/mL;cTnI 0.6 ng/mL	OR	2.79	2.17–3.58	322 cases	5	[12]
cTnT	Above: 0.1–0.25 ug/L	RR	2.7	2.1–3.4	n.a.	12	[42]
cTnT+cTnI	Above: cTnT 0.1–0.2 ng/mL; cTnI:unknown	OR	2.5	2.0–3.1	241 cases	10	[43]
cTnT+cTnI	Above: 0.1–1.5 ng/mL	OR	2.27	1.62–3.16	2,401 total	3	[44]
cTnI	Above: 2.3–0.026 ng/mL	OR	1.77	1.36–2.30	1,174 cases	16	[45]
cTnT	Above: 0.1–0.03 ng/mL	OR	1.77	1.29–2.45	293 cases	6	[45]
cTnT+cTnI	Above: cTnT 0.03–0.1 ng/mL; cTnI 2.3–0.08 ng/mL	OR	1.59	1.29–1.95	6,885 total	15	[46]
***Kidney function***							
Serum creatine (eGFR^9^)	Reference value vs15–29 ml/min/1.73 m2	HR	3.98	3.02–5.24	266,975 total	6	[14]
Cystatin C	Top vs bottom quintile	RR	2.62	2.05–3.37	2,321 cases	13	[11]
Serum creatine (eGFR)	Reference value vs 30–44 ml/min/1.73 m2	HR	2.50	2.10–2.97	266,975 total	6	[14]
Cystatin C	Top vs bottom tertile	RR	1.72	1.27–2.34	741 cases	4	[11]
Serum creatine (eGFR)	Reference value vs 45–59 ml/min/1.73 m2	HR	1.63	1.22–2.18	266,975 total	6	[14]

4 vWF: von Willebrand factor.

5 hs: high sensitivity.

6 cTn: cardiac troponin.

7 BNP: brain natriuretic peptide.

8 NT-pro: amino terminal prohormone of

9 eGFR: estimated glomerular filtration rate.

**Table 3:** Selection of meta-analyses of cohorts without pre-existing cardiovascular disease on markers for stroke.

Marker	Risk Applies To	Risk	Results	95% ci	N Patients	N Cohorts	Reference
***Hemostasis***							
Fibrinogen	1-g/L increase	HR	1.75	1.55–1.98	2,775 cases	31	[5]
***Kidney function***							
Serum uric acid	Above: n.a.	RR	1.47	1.19–1.76	1,031 cases	4	[15]

**Figure 2:**
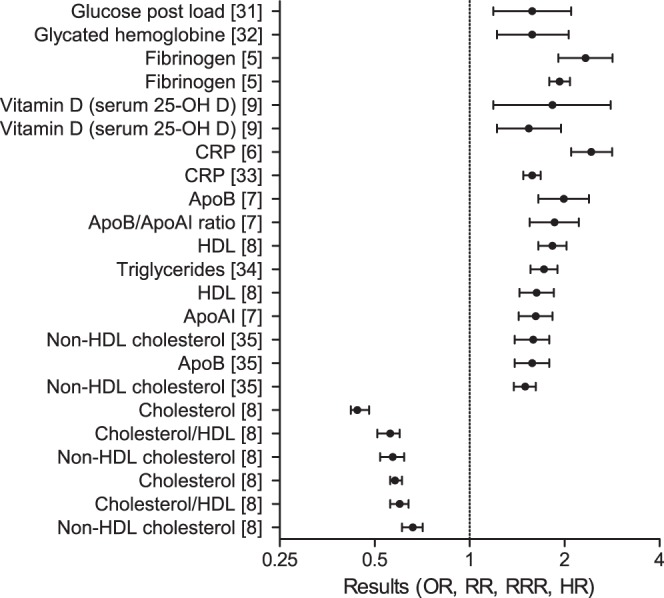
Plot of the results of meta-analyses on CVD events in populations without pre-existing CVD. The results of a selection of meta-analyses on CVD events in populations without pre-existing CVD with a result over 1.5 or under 0.66 are graphically represented. Details of the studies are described in Table 1 and Table S1 in File S1. Abbreviations: CRP: C-reactive protein, Apo: apolipoprotein, HDL: high density lipoprotein.

In total, 61 meta-analyses were found for cardiovascular disease events in populations without pre-existing cardiovascular disease. In these populations, the highest risk for cardiovascular disease is reported for markers associated with hemostasis, inflammation and lipids. These include, from highest to lower result: C-reactive protein (CRP) (RR: 2.43, 95% confidence interval (ci): 2.10–2.83), fibrinogen (HR: 2.33, 95%ci: 1.91–2.84), cholesterol (HR: 0.44, 95%ci: 0.42–0.48), apolipoprotein (Apo) B (RR: 1.99, 95%ci: 1.65–2.39), ApoA/ApoB ratio (RR: 1.86, 95%ci: 1.55–2.22), high density lipoprotein (HDL) (HR: 1.83, 95%ci: 1.65–2.03), and Vitamin D (HR: 1.83, 95%ci: 1.19–2.80) [5]–[9] (Figure 2, Table 1, Table S1 in File S1).

**Figure 3:**
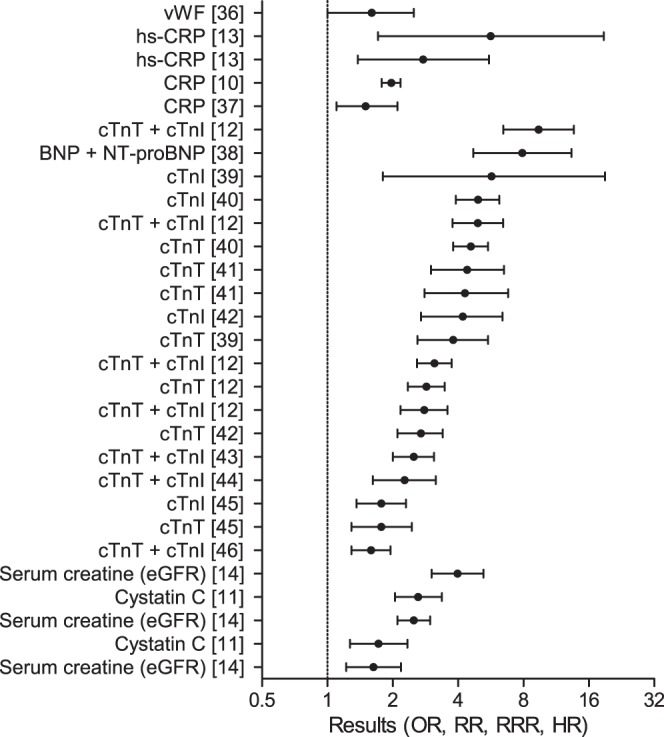
Plot of the results of meta-analyses on CVD events in populations with pre-existing CVD. The results of a selection of meta-analyses on CVD events in populations with pre-existing CVD with a result over 1.5 or under 0.66 are graphically represented. Details of the studies are described in Table 2 and Table S2 in File S1. Abbreviations: vWF: von Willebrand Factor, (hs)-CRP: (high sensitivity) C-reactive protein, cTnT/I: cardiac troponin T/I, (NT-pro)BNP: (amino terminal prohormone of) brain natriuretic peptide, eGFR: estimated glomerular filtration rate.

For populations with pre-existing cardiovascular disease, 43 meta-analyses were found reporting on markers for cardiovascular disease events. Markers with high prognostic value were associated with hemostasis, ischemia, inflammation and kidney function. These include, from highest to lower result: cardiac troponin (cTn) I and T (OR: 9.39, 95%ci: 6.46–13.67), high sensitivity (hs) CRP (OR: 5.65, 95%ci: 1.71–18.73), serum creatinine (HR: 3.98, 95%ci: 3.02–5.24), and cystatin C (RR: 2.62, 95%ci: 2.05–3.37) [10]–[14] (Figure 3, Table 2, Table S2 in File S1).

**Figure 4:**
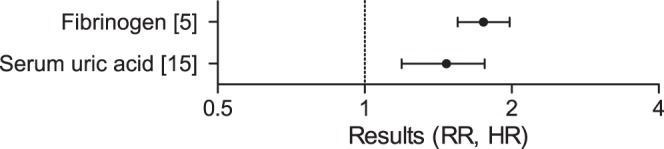
Plot of the results of meta-analyses on stroke events in populations without pre-existing CVD. The results of a selection of meta-analyses on stroke events in populations without pre-existing CVD are graphically represented. Details of the studies are described in Table 3 and Table S3 in File S1.

For ischemic stroke events in individuals without pre-existing cardiovascular disease, 18 meta-analyses were found. These were related to hemostasis and kidney function, being fibrinogen (HR: 1.75, 95%ci: 1.55–1.98), and serum uric acid (RR: 1.47, 95%ci: 1.19–1.76) [5], [15] (Figure 4, Table 3, Table S3 in File S1).

## Discussion

Cardiovascular disease is one of the major causes of death world-wide. Studies that evaluate the predictive value of serological biomarkers for cardiovascular disease have grown in large numbers, which has made it difficult to keep track on the overall predictive value of specific biomarkers. Meta-analyses summarize the results of different cohort studies and present one aggregate score per biomarker, but a general overview presenting the overall results of different biomarkers described in the literature is still lacking. This systematic review of meta-analyses on levels of serological biomarkers for atherothrombosis was performed to provide a comprehensive overview of the state of art, and to compare the relevance of the most commonly studied biomarkers. We conclude that for primary cardiovascular events, markers with strong predictive potential are mainly associated with lipids. For secondary cardiovascular events, markers with strong predictive potential are associated with ischemia. Fibrinogen has strong predictive potential for primary stroke.

The clinical relevance of a marker depends not only on its risk prediction strength, but also on the setup of the investigations (e.g. case-control versus cohort study). The quality of the reporting of results (e.g. reporting of adjustment for other risk factors) is another important aspect. It is attractive to use a score to assess the quality and clinical value of meta-analyses as it gives means to rank the reports. Conversely, a score to assess the quality and clinical value requires assigning weight to different factors that influence the results, which is difficult and hard to motivate. Therefore, we have abstracted aspects of the meta-analyses that may have influenced their results without assigning scores. These aspects, summarized in the methods, were adapted from Hlatky et al., 2009 and are reported in the columns of the tables. These rankings provide the reader with insight into the quality and clinical relevance of the markers.

Many of the markers listed in the tables are well known, and some are established risk markers that are applied in the clinic as a risk marker for cardiovascular disease, for example cholesterol. This review also presents markers with strong predictive value that are not used in the clinic for cardiovascular disease risk prediction, such as fibrinogen, vitamin D, and cystatin C. Such markers, which are associated with high risk but which are without clinical application in cardiovascular disease risk prediction as of yet are of special interest as these may prove to be valuable biomarkers in the future. To have clinical utility, these biomarkers should be able to predict risk independently of other established risk markers. In addition, there should be an established assay that is specific and sensitive in measuring the markers [16], [17]. The possibility to intervene therapeutically based on the levels of risk marker, associated with a reduced risk for cardiovascular disease enables the option to use it to evaluate the efficacy of a therapeutic intervention. With these aspects in mind, we will discuss the clinical utility of fibrinogen, vitamin D, and cystatin C in cardiovascular disease management.

Fibrinogen is one of the strongest markers for both predicting stroke and cardiovascular disease in populations without pre-existing cardiovascular disease. It is involved in hemostasis and blood viscosity. Moreover it is known as an acute phase reactant [18]. Age, sex and cohort corrected results remained significant for cardiovascular disease events and stroke [5].

There are 40 different assays to measure fibrinogen, and although they are reported to be relatively accurate, there is much to gain on assay standardization for overall comparability of measurements [19]. In addition, there is great variation in results between different laboratories, with concentrations ranging from 121 to 437 mg/dL for one specific sample [19]. Improvement in assay standardization would make fibrinogen an interesting biomarker.

Specific members of the fibrate class bezafibrate and clofibrate are able to lower fibrinogen levels besides improving high density lipoprotein and triglyceride levels [20]. However, they have not been shown to be of any benefit in reducing cardiovascular disease risk in relation to their fibrinogen lowering levels [21]. Lowering fibrinogen with bezafibrate also has no effect on occurrence of secondary stroke [22]. A causal relationship of high fibrinogen levels and increased cardiovascular disease risk is unclear, as only some of the polymorphisms that influence the level of fibrinogen are associated with increased cardiovascular disease risk [18]. Two genetic variants that affect the levels of fibrinogen are related to the risk for ischemic stroke, but not for myocardial infarction [23].

Low levels of vitamin D are an independent risk factor for cardiovascular death in populations without pre-existing cardiovascular disease [9]. Systematic reviews on interventional vitamin D supplementation and cardiovascular disease risk reported that vitamin D supplementation had no effect on cardiovascular disease risk, indicating a lack of a causal relationship [24], [25].

Serum vitamin D level is widely measured in diagnostic laboratories to assess vitamin D status in a number of clinical conditions such as rickets, osteomalacia, osteoporosis, hyperparathyroidism, chronic kidney disease or pregnancy [26]. The main type of assays are either competitive immunoassays, or direct detection methods with high performance liquid chromatography or liquid chromatography combined with tandem mass spectrometry [26]. There is considerable variation between the results obtained with the various methods, as well as between laboratories [26]. A standard for vitamin D measurements (SRM 972) is available to increase comparability across laboratories, but as of yet it is unclear how comparability has improved. Immunoassays are less sensitive and specific for vitamin D measurements than high performance liquid chromatography, and liquid chromatography combined with tandem mass spectrometry. The latter two techniques are less attractive in aspects of high throughput and required training of staff [26].

For secondary cardiovascular events, cystatin C is one of the strongest risk predictors. Plasma cystatin C is a marker for chronic kidney disease, a disease strongly associated with an increased risk for cardiovascular disease [27], [28]. The contribution of cystatin C in a multivariate model remains significant, which indicates its added value to established risk factors [11]. The reason for the incremental prognostic information given by cystatin C is still unknown, but it is likely to be related to the sensitivity of cystatin C to detect preclinical kidney dysfunction [28].

Because of the association of renal dysfunction with cardiovascular disease, it is unclear whether cystatin C is a direct marker of cardiovascular disease or merely a marker for renal failure, which has implications for therapeutic intervention. In addition, no therapy has been evaluated to date that aimed to treat patients for cardiovascular disease on stratification by cystatin C values [28]. Cystatin C is measured by immunoassays, using particles coated with cystatin-C specific antibodies, and subsequent turbidometry or nephelometry [29]. The assays are precise, as both detection methods provide coeffients of variation ranging from 2 to 8% [30].

This systematic review is subject to some limitations. This review has included only meta-analyses, so the novelty of reported markers is limited. Also, risk markers are absent in this review when they have not been included in a meta-analysis. Some of the meta-analyses are smaller in size than some single cohort studies. The advantage of a meta-analysis compared with a single large cohort study is that the results represent the ability of a marker to predict events in different cohorts, which increases reliability. Heterogeneity among the meta-analyses exists also in the adjustment for other prognostic factors, and in the methods used to pool the results. This limits the comparability of the different risk markers. Lastly, there is no widely acknowledged search strategy, neither for prognostic studies, nor for meta-analyses. We therefore have applied a broad search strategy, but still some meta-analyses may have been missed.

With cardiovascular disease being one of the major causes of death worldwide, there is an ongoing need for new biomarkers that are able to assist in clinical decision making. Markers such as fibrinogen, vitamin D, and cystatin C have a strong association with cardiovascular disease but as of yet have not been implemented in the clinic. Other emerging types of biomarkers for cardiovascular disease risk prediction may prove their value in the future. A novel initiative in cardiovascular risk prediction is the Circulating Cells Consortium that investigates the information present in circulating cells such as platelets and leukocytes in relation to cardiovascular disease events. As these cells interact with the vessel wall, their responsiveness may convey clinical relevant information on cardiovascular disease risk.

## Supporting Information

File S1 **Tables S1–S9.**
(DOC)
