# Supplementary material for: Circulating Biomarkers for Predicting Cardiovascular Disease Risk; a Systematic Review and Comprehensive Overview of Meta-Analyses
Source: PLoS One. 2013 Apr 22;8(4):e62080. doi: 10.1371/journal.pone.0062080 (PMC3632595; doi:10.1371/journal.pone.0062080)
Supplement: File S1 — Tables S1–S9. (DOC) [file pone.0062080.s001.doc]

*Supplementary Methods*

Terms used for search in databases. The “Outcome” terms were combined with the “Determinant” terms both separated with “[]” brackets, using the command “AND”. Grey marked sections apply only for Embase searches, to exclude results from medline.

**Outcome**: “arterial” OR "arterial ischaemia" OR "arterial ischemia" OR "ischaemic event" OR "ischemic event" OR "arterial occlusion" OR “occlusive event” OR "arterial thrombosis" OR "atherothrombosis" OR “athero thrombosis” OR “athero” OR “vascular” OR “cardiovascular disease” OR “cardio vascular disease” OR “cardiovascular ischaemia” OR “cardiovascular ischemia” OR “cardio vascular ischaemia” OR “cardio vascular ischemia” OR "stroke" OR “ischaemic stroke" OR “ischemic stroke” OR “brain infarction” OR “brain ischaemia” OR “brain ischemia” OR “transient ischaemic attack” OR “transient ischemic attack” OR “heart infarction” OR “myocardial infarction” OR “myo-cardial infarction” OR “myo cardial infarction” OR “myocardial” OR “myo-cardial” OR “myo cardial” OR “coronary artery occlusion” OR “coronary artery ischaemia” OR “coronary artery ischemia” OR “coronary heart disease” OR “coronary artery” OR “atherosclerosis” OR “athero-sclerosis” OR “athero sclerosis” AND [embase]/lim NOT [medline]/lim AND [1988-2012]/py

**Determinant**: “meta analysis” OR “meta-analysis” OR “metaanalysis” OR “systematic review” OR “systematic analysis” OR “quantitative review” OR “quantitative analysis” OR “comparing published studies” OR “combining results” OR “combined results” OR “combining data” OR “combined data” OR “pooling results” OR “pooled results” OR “pooling data” OR “pooled data” AND [embase]/lim NOT [medline]/lim AND [1988-2012]/py

**Table S1. Meta-analyses of cohorts without pre-existing cardiovascular disease on markers for cardiovascular disease risk**.

| **Marker** | **Outcome** | **Risk Applies To** | **Risk** | **Results** | **95% ci[[1]](#footnote-2)** | **N Patients** | **N Cohorts** | **Het.[[2]](#footnote-3)** | **Pooling Method** | **Adj.[[3]](#footnote-4)** | **Patient group** | **IPD[[4]](#footnote-5)** | **Follow-up Period** | **Publication** |
| --- | --- | --- | --- | --- | --- | --- | --- | --- | --- | --- | --- | --- | --- | --- |
|  |  |  |  |  |  |  |  |  |  |  |  |  |  |  |
| ***Diabetes related*** |  |  |  |  |  |  |  |  |  |  |  |  |  |  |
| Glucose (post load) | Fatal, non-fatal CVD[[5]](#footnote-6) | Above: 7.8 mmol/L | RR | 1.58 | 1.19 - 2.10 | 1,467 cases | 7 | no | Random effects model | n.a.[[6]](#footnote-7) | No pre-existing disease | no | 5 -15.6 yr | [1] |
| Glycated hemoglobine (HBA(1c)) | CVD death | HbA1c level: 0.7 | RR | 1.58 | 1.22 - 2.06 | 1,366 cases | 7 | no | Random effects model | yes | No pre-existing disease | no | 9.2 yr mean | [2] |
| Glycated hemoglobine (HBA(1c)) | CVD death | HbA1c level: 0.6 | RR | 1.34 | 1.13 - 1.58 | 1,366 cases | 7 | no | Random effects model | yes | No pre-existing disease | no | 9.2 yr mean | [2] |
| Glucose (fasting) | Fatal, non-fatal CVD | Above 6.1 mmol/L | RR | 1.33 | 1.06 - 1.67 | 1,053 cases | 6 | no | Random effects model | n.a. | No pre-existing disease | no | 4 -14 yr | [1] |
| Glycated hemoglobine (HBA(1c)) | CHD[[7]](#footnote-8) death, non-fatal MI[[8]](#footnote-9) | 1 SD[[9]](#footnote-10) increase | RR | 1.20 | 1.10 - 1.31 | 1,639 cases | 9 | yes | Random effects model | yes | No pre-existing CVD, Western | no | > 1 yr | [3] |
| Glycated hemoglobine (HBA(1c)) | CVD death | HbA1c level: 0.5 | RR | 1.13 | 1.05 - 1.21 | 1,366 cases | 7 | no | Random effects model | yes | No pre-existing disease | no | 9.2 yr mean | [2] |
| Glucose (fasting) | CHD death, non-fatal MI | 1mmol/L increase | RR | 1.06 | 1.00 - 1.12 | 10,808 cases | 23 | yes | Random effects model | yes | No pre-existing CVD, Western | no | > 1 yr | [3] |
| Glucose (non fasting) | CHD death, non-fatal MI | 1mmol/L increase | RR | 1.05 | 1.03 - 1.07 | 12,652 cases | 27 | yes | Random effects model | yes | No pre-existing CVD, Western | no | > 1 yr | [3] |
|  |  |  |  |  |  |  |  |  |  |  |  |  |  |  |
| ***Hemostasis*** |  |  |  |  |  |  |  |  |  |  |  |  |  |  |
| Fibrinogen | Other vascular death | 1 g/L increase | HR | 2.33 | 1.91 - 2.84 | 992 cases | 31 | n.a. | Cox regression | yes | No pre-existing CHD | yes | > 1 yr | [4] |
| Fibrinogen | CHD | 1 g/L increase | HR | 1.93 | 1.79 - 2.08 | 7,118 cases | 31 | yes | Cox regression | yes | No pre-existing CHD | yes | >1 yr | [4] |
| Von Willebrand Factor | Cardiac death, non-fatal MI | Top vs bottom tertile | OR | 1.23 | 1.14 - 1.33 | 3,969 cases | n.a. | n.a. | Inverse variance weighted mean | yes | No pre-existing CVD | no | n.a. | [5] |
|  |  |  |  |  |  |  |  |  |  |  |  |  |  |  |
| ***Hormones*** |  |  |  |  |  |  |  |  |  |  |  |  |  |  |
| Vitamin D (serum 25-OH D) | CVD death | Decrease in different predefined categories | HR | 1.83 | 1.19 - 2.80 | 2,007 cases | 5 | yes | Random effects model | yes | No pre-existing CVD | no | 6.2 - 27.1 yr | [6] |
| Vitamin D (serum 25-OH D) | CVD | Decrease in different predefined categories | HR | 1.54 | 1.22 - 1.95 | 756 cases | 4 | no | Random effects model | yes | No pre-existing CVD | no | 5 - 10 yr | [6] |
| Thyroid stimulating hormone | CHD | Above 4-5 mU/L | RR | 1.18 | 1.02 - 1.38 | 8,071 total | 3 | n.a. | Fixed effects model | yes | No pre-existing CVD | no | 4 - 20 yr | [7] |
| Testosteron | MI, IHD[[10]](#footnote-11), CVD, atherosclerosis, stroke, death | 1 SD increase | RR | 0.89 | 0.83 - 0.96 | 4,598 cases | 18 | yes | Inverse variance weighted mean | yes | No pre-existing vascular disease | no | 3 - 15.3 yr | [8] |
| Testosteron | MI, IHD, stroke, atherosclerosis, death | 1 SD increase | RR | 0.88 | 0.78 - 1.00 | 1,709 cases | 7 | yes | Inverse variance weighted mean | yes | No pre-existing vascular disease | no | 3 - 15.3 yr | [8] |
|  |  |  |  |  |  |  |  |  |  |  |  |  |  |  |
| ***Inflammation*** |  |  |  |  |  |  |  |  |  |  |  |  |  |  |
| CRP[[11]](#footnote-12) | CHD | Top vs bottom tertile | RR | 2.43 | 2.10 - 2.83 | 3,181 cases | 12 | n.a. | Random effects model | n.a. | Healthy individuals | no | 2.9 - 20 yr | [9] |
| CRP | Cardiac death, non-fatal MI | Top vs bottom tertile | OR | 1.58 | 1.48 - 1.68 | 7,068 cases | 22 | yes | Inverse variance weighted mean | yes | No pre-existing CVD | no | 12 yr mean | [5] |
| CRP | CHD death, MI | 1 SD increase | RR | 1.37 | 1.27 - 1.48 | 5,373 cases | 31 | yes | Random effects model | yes | No pre-existing CVD | yes | > 1yr | [10] |
| IL-6 | CHD death, MI | 1 SD increase | OR | 1.26 | 1.19 - 1.35 | 5,730 cases | 17 | yes | Random effects model | yes | No pre-existing vascular disease | no | 6 yr mean | [11] |
|  |  |  |  |  |  |  |  |  |  |  |  |  |  |  |
| ***Kidney function*** |  |  |  |  |  |  |  |  |  |  |  |  |  |  |
| Serum creatine (eGFR)[[12]](#footnote-13) | CHD death, non-fatal MI | Below 60mL/min/1.73m2 | RR | 1.32 | 1.19 - 1.47 | 4,720 cases | 7 | no | Fixed effects model | yes | No pre-existing vascular disease | no | >1 yr | [12] |
| Serum uric acid | CHD death | Above 4.7 - 7.7 mg/dL | RR | 1.16 | 1.01 - 1.30 | 4,038 cases | 8 | yes | Random effects model | yes | No pre-existing CVD | no | 8.2 - 24.9 yr | [13] |
| Serum uric acid | CHD | Above 4.7 - 7.7 mg/dL | RR | 1.09 | 1.03 - 1.16 | 5,198 cases | 9 | yes | Random effects model | yes | No pre-existing CVD | no | 6.5 - 17.5 yr | [13] |
|  |  |  |  |  |  |  |  |  |  |  |  |  |  |  |
| ***Lipids*** |  |  |  |  |  |  |  |  |  |  |  |  |  |  |
| Apo[[13]](#footnote-14)B | CHD | Top vs bottom tertile | RR | 1.99 | 1.65 - 2.39 | 6,920 cases | 19 | yes | Random effects model | yes | No pre-exsting CVD | no | 9 yr mean | [14] |
| ApoB/ApoAI ratio | CHD | Top vs bottom tertile | RR | 1.86 | 1.55 - 2.22 | 3.730 cases | 7 | yes | Random effects model | yes | No pre-existing CVD | no | 10 yr mean | [14] |
| HDL[[14]](#footnote-15) | IHD death | 0.33 mmol/L decrease | HR | 1.83 | 1.65 - 2.03 | 1,198 cases | 23 | no | Cox regression | no | No pre-existing CVD (age 60-69) | yes | 8 yr mean | [15] |
| Triglycerides | CHD death, non-fatal MI | Top vs bottom tertile | OR | 1.72 | 1.56 - 1.90 | 10,158 cases | 29 | yes | Inverse variance weighted mean | yes | No pre-existing disease | no | 3.2 - 20 yr | [16] |
| HDL | IHD death | 0.33 mmol/L decrease | HR | 1.63 | 1.44 - 1.85 | 764 cases | 23 | yes | Cox regression | no | No pre-existing CVD (age 40-59) | yes | 8 yr mean | [15] |
| ApoAI | CHD | Bottom vs top tertile | RR | 1.62 | 1.43 - 1.83 | 6,333 cases | 21 | yes | Random effects model | yes | No pre-exsting CVD | no | 9 yr mean | [14] |
| Non-HDL cholesterol | CHD | 43 mg/dL increase | HR | 1.59 | 1.36 - 1.85 | 12,785 cases | 68 | yes | Random effects model | yes | No pre-existing vascular disease | yes | 8.1 yr median | [17] |
| ApoB | CHD | 29 mg/dL increase | HR | 1.58 | 1.39 - 1.79 | 4,499 cases | 22 | yes | Random effects model | yes | No pre-existing vascular disease | yes | 8.1 yr median | [17] |
| Non-HDL cholesterol | CHD | 1.53 unit increase | HR | 1.50 | 1.38 - 1.62 | 4,499 cases | 22 | yes | Random effects model | yes | No pre-existing vascular disease | yes | 8.1 yr median | [17] |
| ApoB/ApoAI ratio | CHD | 0.27 unit increase | HR | 1.49 | 1.39 - 1.60 | 4,499 cases | 22 | yes | Random effects model | yes | No pre-existing vascular disease | yes | 8.1 yr median | [17] |
| Cholesterol | CVD death | Fourth vs other quartiles | HR | 1.49 | 1.05 - 2.10 | 201 cases | 5 | no | Cox regression | yes | No pre-existing CVD | yes | 4.5 - 10.6 yr | [18] |
| Low densitty lipoprotein | CHD | 33 mg/dL increase | HR | 1.38 | 1.09 - 1.73 | 2,076 cases | 8 | yes | Cox regression | yes | No pre-existing vascular disease | yes | 8.1 yr median | [17] |
| HDL | IHD death | 0.33 mmol/L decrease | HR | 1.35 | 1.22 - 1.49 | 1,058 cases | 23 | no | Cox regression | no | No pre-existing CVD (age 70-89) | yes | 8 yr mean | [15] |
| Cholesterol | CHD death | 1 mmol/L increase | RR | 1.22 | 1.18 - 1.27 | 18,244 total | 6 | n.a. | Fixed effects model | yes | Not selected on any disease, Men; middle-age and above | no | 3 - 32 yr | [19] |
| Cholesterol | CHD death | 1 mmol/L increase | RR | 1.22 | 1.15 - 1.28 | 13,486 total | 11 | n.a. | Fixed effects model | yes | Not selected on any disease, Men: >65 yr | no | 3 - 32 yr | [19] |
| Lipoprotein(a) | CHD | 1 SD increase | RR | 1.10 | 1.02 - 1.18 | 106,645 total | 30 | yes | Random effects model | yes | No pre-exiting CHD | no | >1 yr | [20] |
| Cholesterol | CHD death | 1 mmol/L increase | RR | 1.04 | 0.85 - 1.23 | 9.342 total | 6 | n.a. | Fixed effects model | yes | Not selected on any disease, Women: >65 yr | no | 3 - 32 yr | [19] |
| Triglycerides | CHD | Per 68% increase | HR | 0.99 | 0.94 - 1.05 | 12,785 cases | 68 | yes | Random effects model | yes | No pre-existing vascular disease | yes | 8.1 yr median | [17] |
| Cholesterol | IHD death | 1 mmol/L decrease | HR | 0.85 | 0.82 - 0.89 | 5,626 cases | 61 | no | Cox regression | no | No pre-existing CVD, age 80-89 | yes | 13 yr mean | [15] |
| Cholesterol | IHD death | 1 mmol/L decrease | HR | 0.82 | 0.80 - 0.85 | 10,829 cases | 61 | no | Cox regression | no | No pre-existing CVD, age 70-79 | yes | 13 yr mean | [15] |
| ApoAI | CHD | 29 mg/dL increase | HR | 0.78 | 0.72 - 0.86 | 4,499 cases | 22 | yes | Random effects model | yes | No pre-existing vascular disease | yes | 8.1 yr median | [17] |
| HDL | CHD | 15 mg/dL increase | HR | 0.77 | 0.72 - 0.83 | 12,785 cases | 68 | yes | Random effects model | yes | No pre-existing vascular disease | yes | 8.1 yr median | [17] |
| Non-HDL cholesterol | IHD death | 1 mmol/L decrease | HR | 0.73 | 0.67 - 0.79 | 1,058 cases | 23 | no | Cox regression | no | No pre-existing CVD (age 70-89) | yes | 8 yr mean | [15] |
| Cholesterol | IHD death | 1 mmol/L decrease | HR | 0.72 | 0.69 - 0.74 | 10,419 cases | 61 | no | Cox regression | no | No pre-existing CVD, age 60-69 | yes | 13 yr mean SD:6 | [15] |
| Cholesterol/HDL ratio | IHD death | 1.33 units decrease | HR | 0.69 | 0.63 - 0.74 | 1,058 cases | 23 | no | Cox regression | no | No pre-existing CVD (age 70-89) | yes | 8 yr mean | [15] |
| Non-HDL cholesterol | IHD death | 1 mmol/L decrease | HR | 0.66 | 0.61 - 0.71 | 1,198 cases | 23 | no | Cox regression | no | No pre-existing CVD (age 60-69) | yes | 8 yr mean | [15] |
| Cholesterol/HDL ratio | IHD death | 1.33 units decrease | HR | 0.60 | 0.56 - 0.64 | 1,198 cases | 23 | yes | Cox regression | no | No pre-existing CVD (age 60-69) | yes | 8 yr mean | [15] |
| Cholesterol | IHD death | 1 mmol/L decrease | HR | 0.58 | 0.56 - 0.61 | 5,561 cases | 61 | yes | Cox regression | no | No pre-existing CVD, age 50-59 | yes | 13 yr mean SD:6 | [15] |
| Non-HDL cholesterol | IHD death | 1 mmol/L decrease | HR | 0.57 | 0.52 - 0.62 | 764 cases | 23 | no | Cox regression | no | No pre-existing CVD (age 40-59) | yes | 8 yr mean | [15] |
| Cholesterol/HDL ratio | IHD death | 1.33 units decrease | HR | 0.56 | 0.51 - 0.60 | 764 cases | 23 | no | Cox regression | no | No pre-existing CVD (age 40-59) | yes | 8 yr mean | [15] |
| Cholesterol | IHD death | 1 mmol/L decrease | HR | 0.44 | 0.42 - 0.48 | 1,309 cases | 61 | yes | Cox regression | no | No pre-existing CVD, (age 40-49) | yes | 13 yr mean | [15] |
|  |  |  |  |  |  |  |  |  |  |  |  |  |  |  |
| ***Miscellaneous*** |  |  |  |  |  |  |  |  |  |  |  |  |  |  |
| Homocysteine | Cardiac events | Homocysteine levels increase | RR | 1.38 | 1.16 - 1.63 | 2,529 cases | 15 | yes | Random effects model | yes | No pre-existing CVD | no | 3 - 12.8 yr | [21] |
| Erythrocyte sedimentation rate | Cardiac death, non-fatal MI | Top vs bottom tertile | OR | 1.33 | 1.22 - 1.44 | 4,386 cases | 6 | n.a. | Inverse variance weighted mean | yes | No pre-existing CVD | no | n.a. | [5] |
| Homocysteine | CHD | 5 μmol/L increase | OR | 1.23 | 1.06 -1.41 | 1,943 cases | 10 | yes | Random effects model | no | No pre-existing CVD | no | n.a. | [22] |
| Homocysteine | CHD | 5 μmol/L increase | RR | 1.18 | 1.10 - 1.26 | 22,652 total | 21 | no | Random effects model | yes | No pre-existing vascular disease | no | 2.7 - 24 yr | [23] |
| Homocysteine | CHD | 5 μmol/L increase | OR | 1.06 | 0.99 - 1.13 | 269 cases | 2 | no | Fixed effects model | no | No pre-existing CVD, males | no | n.a. | [22] |
| Homocysteine | IHD | 25% lower usual homocysteine level | OR | 0.89 | 0.83 - 0.96 | 1,855 cases | 11 | yes | Regression | yes | No pre-existing cerebrovascular disease | yes | n.a. | [24] |
| Selenium | CHD, MI | Top vs bottom quantiles | RR | 0.85 | 0.74 - 0.99 | 1,366 cases | 14 | no | Random effects model | yes | No pre-existing CVD | no | 3 - 25 yr | [25] |

**Table S2. Meta-analyses of cohorts with pre-existing cardiovascular disease on markers for cardiovascular disease risk.**

| **Marker** | **Outcome** | **Risk Applies to** | **Risk** | **Results** | **95% ci** | **N Patients** | **N Cohorts** | **Het.** | **Pooling Methods** | **Adj.** | **Patient group, general population** | **IPD** | **follow-up period** | **Publication** |
| --- | --- | --- | --- | --- | --- | --- | --- | --- | --- | --- | --- | --- | --- | --- |
|  |  |  |  |  |  |  |  |  |  |  |  |  |  |  |
| ***Hemostasis*** |  |  |  |  |  |  |  |  |  |  |  |  |  |  |
| Von Willebrand Factor | CHD death, non-fatal MI | Top vs bottom tertile | OR | 1.6 | 1.0 - 2.5 | 723 cases | 8 | no | Inverse variance weighted mean | yes | Pre-existing CVD | no | 7 yr mean | [26] |
| Fibrinogen | Acute coronary events | Above median fibrinogen levels | HR | 1.42 | 1.18 - 1.70 | 477 cases | 3 | no | Cox regression | yes | TIA or ischemic stroke patients | yes | 2.6 - 10 yr | [27] |
| Tissue plasminogen activator | CHD death, non-fatal MI | Top vs bottom tertile | OR | 1.32 | 0.70 - 2.50 | 450 cases | 6 | yes | Inverse variance weighted mean | yes | Pre-existing CVD | no | 8 yr mean | [28] |
| Fibrinogen | Acute ischemic vascular events | Above median fibrinogen levels | HR | 1.31 | 1.15 - 1.49 | 1,005 cases | 3 | no | Cox regression | yes | TIA or ischemic stroke patients | yes | 2.6 - 10 yr | [27] |
|  |  |  |  |  |  |  |  |  |  |  |  |  |  |  |
| ***Inflammation*** |  |  |  |  |  |  |  |  |  |  |  |  |  |  |
| hs[[15]](#footnote-16)-CRP | Cardiac death > 180 days after vascular surgery | 1 mg/L > hs-CRP > 3 mg/L | OR | 5.65 | 1.71 - 18.73 | 477 total | 4 | no | Fixed effects model | n.a. | Vascular surgery patients | no | >180 days | [29] |
| hs-CRP | MACE[[16]](#footnote-17) > 180 days after vascular surgery | 1 mg/L > hs-CRP > 3 mg/L | OR | 2.76 | 1.38 - 5.55 | 386 total | 3 | no | Fixed effects model | n.a. | Vascular surgery patients | no | >180 days | [29] |
| hs-CRP/CRP | MACE <30 days after vascular surgery | High risk vs low risk concentration | OR | 2.58 | 0.42 - 16.01 | 85 total | 2 | no | Fixed effects model | n.a. | Vascular surgery patients | no | <30 days | [29] |
| CRP | Death, non-fatal cardiovascular events | Top vs bottom tertile | RR | 1.97 | 1.78 - 2.17 | 6,485 cases | 83 | yes | Random effects model | yes | Stable coronary disease patients | no | 0.014 - 12 yr | [30] |
| CRP | CHD | Top vs bottom tertile | RR | 1.5 | 1.1 - 2.1 | 604 cases | 3 | no | Inverse variance weighted mean | yes | Pre-existing CVD | no | 8 yr mean | [31] |
| hs-CRP | Non-fatal MI > 180 days after vascular surgery | 1 mg/L > hs-CRP > 3 mg/L | OR | 1.38 | 0.63 - 3.03 | 386 total | 3 | no | Fixed effects model | n.a. | Vascular surgery patients | no | >180 days | [29] |
| IL-6 | Death after stroke, CVD, cancer, other | Unit increase | OR | 1.07 | 1.04 - 1.10 | 1,184 total | 3 | yes | Fixed effects model | yes | Stroke patients | no | 0 - 6 monts | [32] |
| Leukocyte count | MI | 1 E6/L increase | HR | 1.02 | 0.99 - 1.05 | 582 cases | 3 | n.a. | Cox regression | no | PCI[[17]](#footnote-18) patients | no | 1 yr | [33] |
|  |  |  |  |  |  |  |  |  |  |  |  |  |  |  |
| ***Ischemia*** |  |  |  |  |  |  |  |  |  |  |  |  |  |  |
| cTn[[18]](#footnote-19)T + cTnI | MI, death | cTnT above 0.1-0.2 ng/mL, cTnI above 0.1-3.1 ng/mL | OR | 9.39 | 6.46 - 13.67 | 160 cases | 10 | no | Fixed effects model | n.a. | Unstable angina patients | no | 30 days | [34] |
| BNP + NT-pro BNP [[19]](#footnote-20) | CHD death, non-fatal MI | BNP above 116 gp/mL, NT-proBNP above 227.5 pg/mL | OR | 7.9 | 4.7 - 13.3 | 75 cases | 5 | yes | Random effects model | yes | Vascular surgery patients | yes | 30 months | [35] |
| cTnI | MI, death | Above unknown level | RR | 5.7 | 1.8 - 19 | 882 cases | 4 | yes | Fixed effects model | n.a. | Unstable angina pectoris patients | no | 4 weeks | [36] |
| cTnI | MI, death | Above different level per study | OR | 4.94 | 3.9 - 6.2 | 1,168 cases | 13 | n.a. | Regression | n.a. | ACS[[20]](#footnote-21), nSTEMI [[21]](#footnote-22)patients | no | 4 days - 6 months | [37] |
| cTnT + cTnI | MI, death | cTnT above 0.1-0.2 ng/mL, cTnI above 0.1-0.6 ng/mL | OR | 4.93 | 3.77 - 6.45 | 1,602 cases | 16 | yes | Fixed effects model | n.a. | nSTEMI patients | no | 30 days | [34] |
| cTnT | MI, death | Above 0.1-0.2 ng/mL | OR | 4.58 | 3.8 - 5.5 | 1,965 cases | 16 | n.a. | Regression | n.a. | ACS, nSTEMI patients | no | 4 days - 6 months | [37] |
| cTnT | Need for revascularization | Above 0.1-0.2 μg/L | OR | 4.4 | 3.0 - 6.5 | 163 cases | 4 | n.a. | Regression | n.a. | Non-AMI[[22]](#footnote-23) patients | no | hospitalization - 34 months | [38] |
| cTnT | Cardiac death, AMI | Above 0.1-0.2 μg/L | OR | 4.3 | 2.8 - 6.8 | 96 cases | 7 | n.a. | Regression | n.a. | Non-AMI patients | no | hospitalization - 34 months | [38] |
| cTnI | Cardiac death, non-fatal MI | Above 0.03-3.1 μg/L | RR | 4.2 | 2.7 - 6.4 | n.a. | 9 | no | Regression | n.a. | Unstable angina pectoris patients | no | 42 days median | [39] |
| cTnT | MI, death | Above unknown level | RR | 3.8 | 2.6 - 5.5 | 1,292 cases | 12 | yes | Fixed effects model | n.a. | Unstable angina pectoris patients | no | 1 -147 weeks | [36] |
| cTnT + cTnI | MI, death | cTnT above 0.1-0.2 ng/mL, cTnI above 0.1-3.1 ng/mL | OR | 3.11 | 2.59 - 3.74 | 201 cases | 21 | no | Fixed effects model | n.a. | Unstable angina patients | no | 5 months -3 yr | [34] |
| cTnT | MI, death | Above 0.1-0.2 ng/ml | OR | 2.86 | 2.35 - 3.47 | 1,330 cases | 3 | no | Fixed effects model | n.a. | STEMI patients | no | 30 days | [34] |
| cTnT + cTnI | MI, death | cTnT above 0.1-0.2 ng/mL, cTnI above 0.6 ng/mL | OR | 2.79 | 2.17 - 3.58 | 322 cases | 5 | no | Fixed effects model | n.a. | nSTEMI patients | no | 5 months -3 yr | [34] |
| cTnT | Cardiac death, non-fatal MI | Above 0.1-0.25 μg/L | RR | 2.7 | 2.1 - 3.4 | n.a. | 12 | n.a. | Regression | n.a. | Unstable angina pectoris patients | no | 30 days median | [39] |
| cTnT + cTnI | MI, death | cTnT above 0.1-0.2 ng/mL, cTnI above unknown level | OR | 2.5 | 2.0 - 3.1 | 241 cases | 10 | n.a. | Random effects model | no | ACS, non-ST elevated patients | no | 1- 147 weeks | [40] |
| cTnT + cTnI | MI | Above 0.1-1.5 ng/ml | OR | 2.27 | 1.62 - 3.16 | 2,401 total | 3 | n.a. | Regression | n.a. | Patients undergoing PCI | no | 1.5 - 68 months | [41] |
| cTnI | Death, nSTEMI | Above 2.3-0.026 ng/mL | OR | 1.77 | 1.36 - 2.30 | 1,174 cases | 16 | no | Random effects model | n.a. | Post elective PCI patients | no | 3 - 50 months | [42] |
| cTnT | Death, nSTEMI | Above 0.1-0.03 ng/ml | OR | 1.77 | 1.29 - 2.45 | 293 cases | 6 | no | Random effects model | n.a. | Post elective PCI patients | no | 6 - 67 months | [42] |
| cTnT + cTnI | MI, death | cTnT above 0.03-0.1 ng/mL, cTnI above 2.3-0.08 ng/mL | OR | 1.59 | 1.29 - 1.95 | 6,885 total | 15 | n.a. | Fixed effects model | n.a. | Post elective PCI patients | no | 3 - 67 months | [43] |
| cTnT + cTnI | Death, MI, revascularization, angina | Above 0.1-1.5 ng/ml | OR | 1.03 | 0.84 - 1.26 | 1,831 cases | 7 | n.a. | Regression | n.a. | Patients undergoing PCI | no | 1.5 - 77 months | [41] |
|  |  |  |  |  |  |  |  |  |  |  |  |  |  |  |
| ***Kidney function*** |  |  |  |  |  |  |  |  |  |  |  |  |  |  |
| Serum creatine (eGFR) | CVD death | Reference value vs 15-29ml/min/1.73m2 | HR | 3.98 | 3.02 - 5.24 | 266,975 total | 6 | n.a. | Random effects model | yes | Hypertension, pre-exist CVD, diabetes | no | 4.1 yr mean | [44] |
| Cystatin C | CVD | Top vs bottom quintile | RR | 2.62 | 2.05 - 3.37 | 2,321 cases | 13 | yes | Random effects model | yes | High CVD risk population, elderly, CVD patients | no | 1 - 12.8 yr | [45] |
| Serum creatine (eGFR) | CVD death | Reference value vs 30-44ml/min/1.73m2 | HR | 2.50 | 2.10 - 2.97 | 266,975 total | 6 | n.a. | Random effects model | yes | High risk CKD[[23]](#footnote-24) (hypertension, pre-exist CVD, diabetes) | no | 4.1 yr mean | [44] |
| Cystatin C | CHD | Top vs bottom tertile | RR | 1.72 | 1.27 - 2.34 | 741 cases | 4 | yes | Random effects model | yes | High CVD risk population, elderly, CVD patients | no | 3 - 7.4 yr | [45] |
| Serum creatine (eGFR) | CVD death | Reference value vs 45-59ml/min/1.73m2 | HR | 1.63 | 1.22 - 2.18 | 266,975 total | 6 | n.a. | Random effects model | yes | High risk CKD (hypertension, pre-exist CVD, diabetes) | no | 4.1 yr mean | [44] |
| Serum creatine (eGFR) | CVD death | Reference value vs 60-74ml/min/1.73m2 | HR | 1.01 | 0.80 - 1.28 | 266,975 total | 6 | n.a. | Random effects model | yes | High risk CKD (hypertension, pre-exist CVD, diabetes) | no | 4.1 yr mean | [44] |
| Serum creatine (eGFR) | CVD death | Reference value vs 75-89ml/min/1.73m2 | HR | 0.98 | 0.85 - 1.13 | 266,975 total | 6 | n.a. | Random effects model | yes | High risk CKD (hypertension, pre-exist CVD, diabetes) | no | 4.1 yr mean | [44] |
|  |  |  |  |  |  |  |  |  |  |  |  |  |  |  |
| ***Lipids*** |  |  |  |  |  |  |  |  |  |  |  |  |  |  |
| Lipoprotein(a) | CHD death, non-fatal MI | Top vs bottom tertile | RR | 1.3 | 1.1 - 1.6 | 1,392 cases | 9 | no | Inverse variance weighted mean | yes | Pre-existing CHD | no | > 1 yr | [46] |
| Lp-PLA2[[24]](#footnote-25) | Vascular death | 1 SD increase | RR | 1.10 | 0.98 - 1.25 | 186 cases | 5 | n.a. | Random effects model | yes | Ischemic event patients | no | 1.1 yr median | [47] |
| Lp-PLA2 | CHD | 1 SD increase | RR | 1.01 | 0.92 - 1.11 | 708 cases | 6 | n.a. | Random effects model | yes | Ischemic event patients | no | 1.1 yr median | [47] |
|  |  |  |  |  |  |  |  |  |  |  |  |  |  |  |
| ***Miscellaneous*** |  |  |  |  |  |  |  |  |  |  |  |  |  |  |
| Hematocrit | CHD death, non-fatal MI | Top vs bottom tertile | RR | 1.81 | 1.19 - 2.76 | 1,162 cases | 3 | no | Inverse variance weighted mean | yes | Pre-existing CVD | no | 16 yr mean | [48] |

**Table S3. Meta-analyses of cohorts without pre-existing cardiovascular disease on markers for stroke**.

| Marker | Outcome | Risk Applies To | Risk | Results | 95% ci | N Patients | N Cohorts | Het. | Pooling Method | Adj. | Patient group | IPD | Follow-up Period | Publication |
| --- | --- | --- | --- | --- | --- | --- | --- | --- | --- | --- | --- | --- | --- | --- |
|  |  |  |  |  |  |  |  |  |  |  |  |  |  |  |
| ***Hemostasis*** |  |  |  |  |  |  |  |  |  |  |  |  |  |  |
| Fibrinogen | Ischemic Stroke | 1 g/L increase | HR | 1.75 | 1.55 - 1.98 | 2,775 cases | 31 | yes | Cox regression | yes | No pre-existing CHD | yes | > 1yr | [4] |
|  |  |  |  |  |  |  |  |  |  |  |  |  |  |  |
| ***Inflammation*** |  |  |  |  |  |  |  |  |  |  |  |  |  |  |
| CRP | Ischemic stroke | Per 1 SD increase | RR | 1.27 | 1.15 - 1.40 | 1,931 cases | 15 | no | Random effects model | yes | No pre-existing CVD | yes | > 1yr | [10] |
|  |  |  |  |  |  |  |  |  |  |  |  |  |  |  |
| ***Kidney function*** |  |  |  |  |  |  |  |  |  |  |  |  |  |  |
| Serum uric acid | Stroke | Above unknown level | RR | 1.47 | 1.19 - 1.76 | 1,031 cases | 4 | no | Random effects model | yes | No previous stroke | no | 7.2 - 12.6 yr | [49] |
| Serum uric acid | Stroke death | Above unknown level | RR | 1.26 | 1.12 - 1.39 | 2,059 cases | 6 | no | Random effects model | yes | No previous stroke | no | 12.4 - 23 yr | [49] |
|  |  |  |  |  |  |  |  |  |  |  |  |  |  |  |
| ***Lipids*** |  |  |  |  |  |  |  |  |  |  |  |  |  |  |
| ApoB | Ischemic stroke | 29 mg/dL increase | HR | 1.19 | 1.05 - 1.34 | 1,192 cases | 8 | yes | Random effects model | yes | No pre-existing vascular disease | yes | 8.1 yr median | [17] |
| non-HDL cholesterol/ HDL | Ischemic stroke | 1.53 unit increase | HR | 1.14 | 1.05 - 1.24 | 1,192 cases | 8 | yes | Random effects model | yes | No pre-existing vascular disease | yes | 8.1 yr median | [17] |
| ApoB/ApoAI ratio | Ischemic stroke | 0.27 unit increase | HR | 1.13 | 1.05 - 1.21 | 1,192 cases | 8 | yes | Random effects model | yes | No pre-existing vascular disease | yes | 8.1 yr median | [17] |
| Cholesterol | ischemic stroke | 1 mmol/L decrease | HR | 1.09 | 0.95 - 1.26 | 225 cases | 61 | yes | Cox regression | no | No pre-existing CVD, age 80-89 | yes | 13 yr mean | [15] |
| Lipoprotein(a) | Ischemic stroke | 1 SD increase | RR | 1.08 | 1.01 - 1.16 | 69,539 total | 13 | no | Fixed effects model | yes | No pre-existing CHD | no | >1 yr | [20] |
| non-HDL cholesterol | Ischemic stroke | 43 mg/dL increase | HR | 1.08 | 0.97 - 1.20 | 2,534 cases | 68 | yes | Random effects model | yes | No pre-existing vascular disease | yes | 8.1 yr median | [17] |
| Cholesterol | Ischemic stroke | 1 mmol/L decrease | HR | 1.06 | 0.95 - 1.17 | 540 cases | 61 | yes | Cox regression | no | No pre-existing CVD, age 70-79 | yes | 13 yr mean | [15] |
| Triglycerides | Ischemic stroke | Per 68% increase | HR | 1.02 | 0.94 - 1.11 | 2,534 cases | 68 | yes | Random effects model | yes | No pre-existing vascular disease | yes | 8.1 yr median | [17] |
| ApoAI | Ischemic stroke | 29 mg/dL increase | HR | 0.97 | 0.88 - 1.08 | 1,192 cases | 8 | yes | Random effects model | yes | No pre-existing vascular disease | yes | 8.1 yr median | [17] |
| HDL | Ischemic stroke | 15 mg/dL increase | HR | 0.96 | 0.90 - 1.02 | 2,534 cases | 68 | yes | Random effects model | yes | No pre-existing vascular disease | yes | 8.1 yr median | [17] |
| Cholesterol | Ischemic stroke | 1 mmol/L decrease | HR | 0.89 | 0.79 - 1.01 | 850 cases | 61 | yes | Cox regression | no | No pre-existing CVD, age 60-69 | yes | 13 yr mean | [15] |
| Cholesterol | Ischemic stroke | 1 mmol/L decrease | HR | 0.73 | 0.61 - 0.87 | 225 cases | 61 | yes | Cox regression | no | No pre-existing CVD, age 40-59 | yes | 13 yr mean | [15] |
|  |  |  |  |  |  |  |  |  |  |  |  |  |  |  |
| ***Miscellaneous*** |  |  |  |  |  |  |  |  |  |  |  |  |  |  |
| Homocysteine | Ischemic stroke | Homocysteine levels increase | RR | 1.37 | 0.99 - 1.91 | 314 cases | 3 | no | Fixed effects model | yes | No pre-existing cerebrovascular disease | no | 5 -12.8 yr | [21] |
| Homocysteine | Stroke | 25% lower usual homocysteine level | OR | 0.81 | 0.69 - 0.95 | 435 cases | 9 | no | Regression | yes | No pre-existing cerebrovascular disease | yes | n.a. | [24] |

**Table S4. Meta-analyses of cohorts with pre-existing cardiovascular disease for stroke.**

| Marker | Outcome | Risk Applies To | Risk | Results | 95% ci | N Patients | N Cohorts | Het. | Pooling Method | Adj. | Patient group | IPD | Follow-up Period | Publication |
| --- | --- | --- | --- | --- | --- | --- | --- | --- | --- | --- | --- | --- | --- | --- |
|  |  |  |  |  |  |  |  |  |  |  |  |  |  |  |
| ***Hemostasis*** |  |  |  |  |  |  |  |  |  |  |  |  |  |  |
| Fibrinogen | Ischemic Stroke | Above median fibrinogen levels (per study) | HR | 1.21 | 1.01 - 1.44 | 512 cases | 3 | no | Cox regression | yes | TIA[[25]](#footnote-26) or ischemic stroke patients | yes | 2.6 - 10 yr | [27] |
|  |  |  |  |  |  |  |  |  |  |  |  |  |  |  |
| ***Lipids*** |  |  |  |  |  |  |  |  |  |  |  |  |  |  |
| Lp-PLA2 | Stroke | 1 SD increase | RR | 1.02 | 0.82 - 1.27 | 111 cases | 4 | n.a. | Random effects model | yes | Ischemic event patients | no | 1.1 yr median | [47] |

**Table S5. Meta-analyses of cohorts representing the general population on markers for cardiovascular disease**.

| **Marker** | **Outcome** | **Risk Applies To** | **Risk** | **Results** | **95% ci** | **N Patients** | **N Cohorts** | **Het.** | **Pooling method** | **Adj.** | **Patient group** | **IPD** | **Follow-up period** | **Publication** |
| --- | --- | --- | --- | --- | --- | --- | --- | --- | --- | --- | --- | --- | --- | --- |
|  |  |  |  |  |  |  |  |  |  |  |  |  |  |  |
| ***Diabetes related*** |  |  |  |  |  |  |  |  |  |  |  |  |  |  |
| Insulin fasting | CVD death | Top vs bottom quantile | HR | 2.66 | 1.45 - 4.90 | 68 cases | 7 | no | Fixed effects model | yes | Population based, no diabetes, women Europe | yes | 6.3 - 11.8 yr | [50] |
| Pro-insulin | CHD death, non-fatal MI | Top vs bottom tertile | OR | 2.23 | 1.65 - 3.00 | 413 cases | 3 | no | Fixed effects model | yes | Population based | no | 9.5 yr mean | [51] |
| Insulin fasting | CVD death | Top vs bottom quantile | HR | 1.54 | 1.16 - 2.03 | 362 cases | 10 | no | Fixed effects model | yes | Population based, no diabetes, men Europe | yes | 4.7 - 12 yr | [50] |
| Insulin post glucose load | CVD death | Top vs bottom quartile | HR | 1.36 | 0.53 - 3.45 | 33 cases | 7 | no | Fixed effects model | yes | Population based, no diabetes, women Europe | yes | 6.3 - 11.8 yr | [50] |
| Insulin non-fasting | CHD death, non-fatal MI | Top vs bottom tertile | OR | 1.35 | 1.14 - 1.60 | 1,980 cases | 8 | no | Fixed effects model | yes | Population based | no | 13.7 yr mean | [51] |
| Insulin non-fating | CHD death, MI, HF[[26]](#footnote-27) | 250 pmol/L increase | RR | 1.25 | 1.03 - 1.51 | 907 cases | 17 | yes | Random effects model | yes | General population | no | 3.5 - 17 yr | [52] |
| Insulin fasting | CHD death, MI, HF | 50 pmol/L increase | RR | 1.17 | 1.09 - 1.26 | 731 cases | 17 | no | Fixed effects model | yes | General population | no | 3.5 - 17 yr | [52] |
| Insulin fasting | CHD death, non-fatal MI | Top vs bottom tertile | OR | 1.12 | 0.98 - 1.28 | 2,649 cases | 14 | yes | Fixed effects model | yes | Population based | no | 9.1 yr mean | [51] |
| Insulin post glucose load | CVD death | Top vs bottom quartile | HR | 0.85 | 0.60 - 1.21 | 295 cases | 10 | no | Fixed effects model | yes | Population based, no diabetes, men Europe | yes | 4.7 - 12 yr | [50] |
|  |  |  |  |  |  |  |  |  |  |  |  |  |  |  |
| ***Hemostasis*** |  |  |  |  |  |  |  |  |  |  |  |  |  |  |
| Fibrinogen | CVD | Top vs bottom tertile | OR | 2.46 | 2.22 - 2.72 | 1,910 cases | 8 | no | Inverse variance weighted mean | no | General population | no | 0.5 - 13.5 yr | [53] |
| Fibrinogen | CVD | Top vs bottom tertile | OR | 2.3 | 1.9 - 2.8 | 15,688 total | 6 | n.a. | Fixed effects model | yes | General population, men mainly | no | ≥2yr | [54] |
| Tissue plasminogen activator | CHD death, non-fatal MI | Top vs bottom tertile | OR | 1.47 | 1.19 - 1.81 | 1,669 cases | 7 | yes | Inverse variance weighted mean | yes | Population based | no | 8 yr mean | [28] |
| Von Willebrand Factor | CHD death, non-fatal MI | Top vs bottom tertile | OR | 1.2 | 0.8 - 1.9 | 899 cases | 6 | no | Inverse variance weighted mean | yes | Population based | no | 7 yr mean | [26] |
| Plasminogen activator inhibitor-1 | CHD death, non-fatal MI | Top vs bottom tertile | OR | 0.98 | 0.53 - 1.81 | 833 cases | 5 | no | Inverse variance weighted mean | yes | Population based | no | 5 yr mean | [28] |
|  |  |  |  |  |  |  |  |  |  |  |  |  |  |  |
| ***Hormones*** |  |  |  |  |  |  |  |  |  |  |  |  |  |  |
| Thyroid stimulating hormone | IHD | Below: 0.1– 0.6 (unit n.a.) | OR | 1.27 | 0.95 - 1.69 | 9,627 total | 5 | yes | Random effects model | yes | Population based | no | 4 -20 yr | [55] |
| Thyroid stimulating hormone | CHD | Above: 4.5 mU/L | RR | 1.21 | 0.88 - 1.68 | 1,392 cases | 5 | no | Random effects model | yes | Population based | no | 2 - 20 yr | [56] |
| Thyroid stimulating hormone | CHD | Below: 4.5 mU/L | RR | 1.20 | 0.97 - 1.49 | 2,134 cases | 10 | no | Random effects model | yes | Population based | no | 2 - 20 yr | [56] |
| Thyroid stimulating hormone | CVD death | Above: 4.5 mU/L | RR | 1.19 | 0.81 - 1.76 | 911 cases | 5 | no | Random effects model | yes | Population based | no | 2 - 20 yr | [56] |
| Thyroid stimulating hormone | CVD death | Below: 4.5 mU/L | RR | 1.18 | 0.98 - 1.42 | 1,167 cases | 8 | no | Random effects model | yes | Population based | no | 2 - 20 yr | [56] |
| Thyroid stimulating hormone | CVD death | Below: 0.1–0.6  (unit n.a.) | OR | 1.09 | 0.84 - 1.41 | 14,719 total | 8 | yes | Random effects model | yes | Population based | no | 4 -20 yr | [55] |
|  |  |  |  |  |  |  |  |  |  |  |  |  |  |  |
| ***Inflammation*** |  |  |  |  |  |  |  |  |  |  |  |  |  |  |
| CRP | CHD | Top vs bottom tertile | RR | 2.0 | 1.6 - 2.5 | 1,953 total | 11 | no | Inverse variance weighted mean | yes | Population based | no | 8 yr mean | [31] |
| Albumin | CHD | Bottom vs top tertile | RR | 1.5 | 1.3 - 1.7 | 3,770 cases | 7 | no | Inverse variance weighted mean | yes | General population | no | 12 yr mean | [57] |
|  |  |  |  |  |  |  |  |  |  |  |  |  |  |  |
| ***Lipids*** |  |  |  |  |  |  |  |  |  |  |  |  |  |  |
| Lipoprotein(a) | CHD death, non-fatal MI | Top vs bottom tertile | RR | 1.7 | 1.4 - 1.9 | 4,044 cases | 18 | no | Inverse variance weighted mean | yes | Population based | no | > 1 yr | [46] |
| Triglycerides | CHD | 1 SD increase | HR | 1.56 | 1.20 - 2.03 | 368 cases | 11 | no | Cox regression | yes | Asia-Pacific population | yes | 2.5 - 19.7 yr | [58] |
| Triglycerides | CHD death | Increase: <0.8, 0.8-1.0, 1.1-1.3, 1.4-1.8, >1.8 mmol/L | HR | 1.38 | 1.18 - 1.62 | 2,082 cases | 30 | yes | Cox regression | yes | Asia-Pacific population | yes | 2.5 - 25.1 yr | [59] |
| Triglycerides | CVD | 1 mmol/L increase | RR | 1.37 | 1.13 - 1.66 | 439 cases | 5 | n.a. | Regression | yes | Population based, women | no | 11.4 yr mean | [60] |
| Cholesterol | CHD death | Increase: <4.2 , 4.2-4.6, 4.7-5.1, 5.2-5.8, >5.8 mmol/L | HR | 1.31 | 1.23 - 1.39 | 2,082 cases | 30 | no | Cox regression | yes | Asia-Pacific population | yes | 2.5 - 25.1 yr | [59] |
| Cholesterol | CHD death | 0.7 mmol/L increase in usual cholesterol level | HR | 1.23 | 1.18 - 1.29 | 4,841 cases | 25 | no | Cox regression | yes | Not selected on any disease, Asia Pacific region | yes | 2.5 - 24.6 yr | [61] |
| Triglycerides | CVD | 1 mmol/L increase | RR | 1.14 | 1.05 - 1.28 | 2,445 cases | 16 | n.a. | Regression | yes | Population based, men | no | 8.4 yr mean | [60] |
|  |  |  |  |  |  |  |  |  |  |  |  |  |  |  |
| ***Miscellaneous*** |  |  |  |  |  |  |  |  |  |  |  |  |  |  |
| Erythrocyte sedimentation rate | CHD death | Top vs bottom tertile | RR | 1.33 | 1.15 - 1.54 | 1,703 cases | 4 | no | Inverse variance weighted mean | yes | Population based | no | 14 yr mean | [48] |
| Hematocrit | CHD death, non-fatal MI | Top vs bottom tertile | RR | 1.16 | 1.05 - 1.29 | 8,020 cases | 16 | no | Inverse variance weighted mean | yes | Population based | no | 16 yr mean | [48] |

**Table S6. Meta-analyses of cohorts with and without cardiovascular disease on markers for cardiovascular disease**.

| **Marker** | **Outcome** | **Risk Applies To** | **Risk** | **Results** | **95% ci** | **N Patients** | **N Cohorts** | **Het.** | **Pooling Method** | **Adj.** | **Patient group** | **IPD** | **follow-up period** | **Publication** |
| --- | --- | --- | --- | --- | --- | --- | --- | --- | --- | --- | --- | --- | --- | --- |
|  |  |  |  |  |  |  |  |  |  |  |  |  |  |  |
| ***Diabetes related*** |  |  |  |  |  |  |  |  |  |  |  |  |  |  |
| Glucose (non fasting) | Fatal, non-fatal CVD | Top vs bottom category | RR | 1.84 | 1.45 - 2.33 | 37,117 total | 5 | n.a. | Random effects model | no | Non-diabetic patients, no exclusion on disease | no | 4.8 - 20 yr | [62] |
| Glycated hemoglobine (HBA(1c)) | Fatal, non-fatal CVD | Top vs bottom category | RR | 1.70 | 0.99 - 2.94 | 3,602 total | 3 | n.a. | Random effects model | yes | Non-diabetic patients, no exclusion on disease | no | 8 yr | [62] |
| Glucose (fasting) | Fatal, non-fatal CVD | Top vs bottom category | RR | 1.27 | 1.13 - 1.43 | 127,617 total | 18 | n.a. | Random effects model | yes | Non-diabetic patients, no exclusion on disease | no | 4 - 22 yr | [62] |
| Glucose post load | Fatal, non-fatal CVD | Top vs bottom category | RR | 1.27 | 1.09 - 1.48 | 61,773 total | 13 | n.a. | Random effects model | yes | Non-diabetic patients, no exclusion on disease | no | 5 -22 yr | [62] |
|  |  |  |  |  |  |  |  |  |  |  |  |  |  |  |
| ***Hemostasis*** |  |  |  |  |  |  |  |  |  |  |  |  |  |  |
| Fibrinogen | CVD | Top vs bottom tertile | OR | 2.35 | 2.14 - 2.57 | 2,581 cases | 13 | no | Inverse variance weighted mean | no | General population + pre-existing CVD | no | 0.5 - 13.5 yr | [53] |
| Fibrinogen | CHD | Top vs bottom tertile | RR | 1.8 | 1.6 - 2.0 | 4,018 cases | 18 | no | Inverse variance weighted mean | yes | General population + pre-existing CVD | no | 8 yr mean | [57] |
| D-dimer fibrinogen | CHD | Top vs bottom tertile | OR | 1.7 | 1.3 - 2.2 | 1,535 cases | 7 | no | Regression | yes | Population based + pre-existing CVD | no | 5 yr mean | [63] |
|  |  |  |  |  |  |  |  |  |  |  |  |  |  |  |
| ***Hormone*** |  |  |  |  |  |  |  |  |  |  |  |  |  |  |
| Adiponectin | CHD death, non-fatal MI | Top vs bottom tertile | OR | 0.84 | 0.70 - 1.01 | 1,313 cases | 7 | no | Inverse variance weighted mean | n.a. | Population based + pre-existing CVD | no | 9.7 yr mean | [64] |
|  |  |  |  |  |  |  |  |  |  |  |  |  |  |  |
| ***Infection*** |  |  |  |  |  |  |  |  |  |  |  |  |  |  |
| *Chlamydia Pneumoniae* IgA titers | CHD | Top vs bottom tertile | OR | 1.25 | 1.03 - 1.53 | 2,283 cases | 10 | no | Inverse variance weighted mean | yes | Population based and pre-existing CVD | no | 11 yr mean | [65] |
| *Chlamydia Pneumoniae* IgG titers | CHD | Top vs bottom tertile | OR | 1.15 | 0.97 - 1.36 | 3,169 cases | 15 | no | Regression | yes | Population based + pre-existing CVD | no | 10 yr mean | [66] |
|  |  |  |  |  |  |  |  |  |  |  |  |  |  |  |
| ***Inflammation*** |  |  |  |  |  |  |  |  |  |  |  |  |  |  |
| CRP | CHD | Top vs bottom tertile | RR | 1.7 | 1.4 - 2.1 | 1,053 cases | 7 | no | Inverse variance weighted mean | yes | General population + pre-existing CVD | no | 6 yr mean | [57] |
| Serum ameloid A | CHD | Top vs bottom tertile | RR | 1.6 | 1.1 - 2.2 | 1,057 cases | 4 | no | Inverse variance weighted mean | yes | Population based + pre-existing CVD | no | 10 yr mean | [31] |
| CRP | CHD death, non-fatal MI | 1.0 mg/mL > CRP > 3.0 mg/mL | RR | 1.58 | 1.37 - 1.83 | 45,563 total | 10 | no | Random effects model | yes | No pre-existing CVD, pre-existing CVD | no | 2.9 - 13 yr | [67] |
| Granulocyte count | CHD death, non-fatal MI | Top vs bottom tertile | RR | 1.51 | 0.99 - 2.30 | 1,643 cases | 5 | yes | Random effects model | yes | Pre-existing CVD, no pre-existing CVD | no | 3 - 18 yr | [68] |
| Leukocyte count | CHD | Top vs bottom tertile | RR | 1.5 | 1.4 - 1.6 | 7229 cases | 19 | yes | Inverse variance weighted mean | yes | General population+ Pre-existing CVD | no | 8 yr mean | [57] |
| Neutrophil count | CHD death, non-fatal MI | Top vs bottom tertile | RR | 1.48 | 1.02 - 2.15 | 1,562 cases | 5 | yes | Random effects model | yes | Pre-existing CVD, no pre-existing CVD | no | 3 - 18 yr | [68] |
| Soluble ICAM[[27]](#footnote-28)1 | CHD death, non-fatal MI | Top vs bottom tertile | OR | 1.39 | 1.11 - 1.73 | 1396 cases | 5 | yes | Inverse variance weighted mean | yes | Population based + pre-existing CVD | no | 11 yr mean | [69] |
| IL-18 | CHD, stroke | Top vs bottom tertile | RR | 1.35 | 1.25 - 1.51 | 3,047 cases | 12 | no | Fixed effects model | yes | Population based, CAD[[28]](#footnote-29) patients, high risk CHD, men | no | 1.58 - 20 yr | [70] |
| Soluble E-Selectin | CHD death, non-fatal MI | Top vs bottom tertile | OR | 1.16 | 0.87 - 1.55 | 832 cases | 2 | no | Inverse variance weighted mean | yes | Population based + pre-existing CVD | no | 13 yr mean | [69] |
| Lymphocyte count | CHD death, non-fatal MI | Top vs bottom tertile | RR | 1.11 | 0.99 - 1.25 | 1,755 cases | 7 | no | Fixed effects model | yes | Pre-existing CVD, no pre-existing CVD | no | 3 - 18 yr | [68] |
| Monocyte count | CHD death, non-fatal MI | Top vs bottom tertile | RR | 1.10 | 0.98 - 1.24 | 1,750 cases | 7 | no | Fixed effects model | yes | Pre-existing CVD, no pre-existing CVD | no | 3 - 18 yr | [68] |
| Soluble VCAM[[29]](#footnote-30)1 | CHD death, non-fatal MI | Top vs bottom tertile | OR | 1.02 | 0.81 - 1.29 | 1307 cases | 4 | no | Inverse variance weighted mean | yes | Population based + pre-existing CVD | no | 11 yr mean | [69] |
|  |  |  |  |  |  |  |  |  |  |  |  |  |  |  |
| ***Ischemia*** |  |  |  |  |  |  |  |  |  |  |  |  |  |  |
| BNP + NT-proBNP | CHD death, non-fatal MI | Top vs bottom tertile | RR | 2.03 | 1.54 - 2.66 | 4,301 cases | 19 | yes | Random effects model | yes | Population based, high CVD risk, pre-existing CVD | no | 5 yr mean | [71] |
|  |  |  |  |  |  |  |  |  |  |  |  |  |  |  |
| ***Kidney function*** |  |  |  |  |  |  |  |  |  |  |  |  |  |  |
| Serum uric acid | CHD | Top vs bottom tertile | OR | 1.22 | 1.05 - 1.40 | 1,645 cases | 8 | yes | Fixed effects model | yes | (No) pre-existing CVD, women | no | 10.5 yr mean | [72] |
| Serum uric acid | CHD | Top vs bottom tertile | OR | 1.12 | 1.05 - 1.19 | 7,813 cases | 15 | yes | Fixed effects model | yes | (No) pre-existing CVD, men | no | 10.5 yr mean | [72] |
|  |  |  |  |  |  |  |  |  |  |  |  |  |  |  |
| ***Lipids*** |  |  |  |  |  |  |  |  |  |  |  |  |  |  |
| Lp-PLA2 | CVD | Top vs bottom quantile | OR | 1.60 | 1.27 - 2.00 | 12,098 total | 8 | yes | Random effects model | yes | No exclusion on disease | no | 1 - 14 yr | [73] |
| ApoB | Fatal, non-fatal CVD | 1 SD increase | RRR | 1.43 | 1.35 - 1.51 | 22,950 cases | 12 | yes | Random effects model | yes | No specified population | no | n.a. | [74] |
| Non-HDL cholesterol | Fatal, non-fatal CVD | 1 SD increase | RRR | 1.34 | 1.24 - 1.44 | 22,950 cases | 12 | yes | Random effects model | yes | No specified population | no | n.a. | [74] |
| LDL[[30]](#footnote-31) | Fatal, non-fatal CVD | 1 SD increase | RRR | 1.25 | 1.18 - 1.33 | 22,950 cases | 11 | yes | Random effects model | yes | No specified population | no | n.a. | [74] |
| Lp-PLA2 | Vascular death | 1 SD increase | RR | 1.13 | 1.05 - 1.22 | 2,887 cases | 11 | no | Random effects model | yes | No pre-existing CVD, stable CHD | no | 5.8 yr median | [47] |
| Lp-PLA2 | CHD | 1 SD increase | RR | 1.11 | 1.08 - 1.15 | 4,361 cases | 12 | no | Fixed effects model | yes | No pre-existing CVD, stable CHD | no | 5.8 yr median | [47] |
|  |  |  |  |  |  |  |  |  |  |  |  |  |  |  |
| ***Miscellaneous*** |  |  |  |  |  |  |  |  |  |  |  |  |  |  |
| Homocysteine | CVD and stroke | Above: 14.5-23.9 μmol/L | OR | 3.74 | 2.53 - 5.54 | 317 cases | 4 | yes | Fixed effects model | no | No exclusion on disease | no | 2.7 - 9 (2 studies n.a.) | [75] |
| Homocysteine | CHD | 5 μmol/L increase | RR | 1.3 | 1.1 - 1.5 | 1,041 cases | 5 | yes | Inverse variance weighted mean | yes | No pre-existing CVD, pre-existing CVD | no | 8 yr mean | [76] |
| Homocysteine | Cardiac death, non-fatal MI | 5 μmol/L increase | OR | 1.23 | 1.14 - 1.32 | 3,144 cases | 16 | n.a. | Random effects model | yes | No renal disease | no | 3 - 13 yr | [77] |
| Serum ferritin | CHD | Above: 200 μg/L | RR | 1.03 | 0.83 - 1.29 | 570 cases | 5 | yes | Inverse variance weighted mean | yes | Population based + pre-existing CVD | no | 8 yr mean | [78] |
| Iron-binding capacity | CHD | Top vs bottom tertile | RR | 0.98 | 0.66 - 1.46 | 2755 cases | 4 | no | Inverse variance weighted mean | yes | Population based + pre-existing CVD | no | 13 yr mean | [78] |
| Transferrin saturation | CHD | Top vs bottom tertile | RR | 0.92 | 0.74 - 1.14 | 6194 cases | 5 | no | Inverse variance weighted mean | yes | Population based + pre-existing CVD | no | 14 yr mean | [78] |
| Serum ferritin | CHD | Top vs bottom tertile | RR | 0.83 | 0.67 - 1.03 | 2848 cases | 3 | yes | Inverse variance weighted mean | yes | Population based + pre-existing CVD | no | 14 yr mean | [78] |

**Table S7. Meta-analyses of miscellaneous cohorts on markers for cardiovascular disease**.

| Marker | Outcome | Risk Applies To | Risk | Results | 95% ci | N Patients | N Cohorts | Het. | Pooling Method | Adj. | Patient group, general population | IPD | Follow-up Period | Publication |
| --- | --- | --- | --- | --- | --- | --- | --- | --- | --- | --- | --- | --- | --- | --- |
|  |  |  |  |  |  |  |  |  |  |  |  |  |  |  |
| ***Ischemia*** |  |  |  |  |  |  |  |  |  |  |  |  |  |  |
| BNP + NT-proBNP | Non-fatal MI | NT-proBNP above 280-533 pg/mL, BNP above 40-100 pg/mL | OR | 6.24 | 1.82 - 21.40 | 33 cases | 3 | no | Fixed effects model | n.a. | Vascular and non cardiac surgery patients | no | <30 days | [79] |
| BNP + NT-proBNP | MACE | NT-proBNP above 280-319 pg/mL, BNP above 35 pg/mL | OR | 3.31 | 2.10 - 5.24 | 95 cases | 2 | yes | Random effects model | n.a. | Vascular and non cardiac surgery patients | no | <180 days | [79] |
| BNP | MACE | Above 35-255 pg/mL | OR | 25.45 | 12.46 - 51.97 | 148 casss | 6 | no | Random effects model | no | Vascular and non-cardiac surgery patients | no | up to 43 days | [80] |
| BNP + NT-proBNP | Cardiac death | NT-proBNP above 201-791 pg/mL, BNP above 35-255 pg/mL | OR | 23.88 | 9.43 - 60.43 | 45 cases | 7 | no | Random effects model | no | Vascular and non-cardiac surgery patients | no | up to 43 days | [80] |
| BNP + NT-proBNP | MACE | NT-proBNP above 280-533 pg/mL, BNP above 40–100 pg/mL | OR | 17.37 | 3.31 - 91.15 | 52 cases | 4 | yes | Random effects model | n.a. | Vascular and non cardiac surgery patients | no | <30days | [79] |
| NT-proBNP | MACE | Above 201-791 pg/mL | OR | 15.65 | 10.39 - 25.37 | 135 cases | 4 | no | Random effects model | no | Vascular and non-cardiac surgery patients | no | up to 43 days | [80] |
|  |  |  |  |  |  |  |  |  |  |  |  |  |  |  |
| ***Kidney function*** |  |  |  |  |  |  |  |  |  |  |  |  |  |  |
| Serum creatine (eGFR) | CVD death | 30 μmol/L decrease | HR | 1.27 | 1.11 - 1.46 | 1,784 cases | 8 | no | Cox regression | yes | Hypertensive patients | yes | 5 yr mean | [81] |
| Serum Uric Acid | CVD death | Above 75 mmol/L | HR | 1.13 | 1.05 - 1.22 | 1,784 cases | 8 | yes | Cox regression | yes | Hypertensive patients | yes | 5 yr mean | [81] |
|  |  |  |  |  |  |  |  |  |  |  |  |  |  |  |
| ***Lipids*** |  |  |  |  |  |  |  |  |  |  |  |  |  |  |
| Cholesterol | CVD death | 1 mmol/L increase | HR | 1.11 | 1.05 - 1.17 | 1,784 cases | 8 | no | Cox regression | yes | Hypertensive patients | yes | 5 yr mean | [81] |

**Table S8. Meta-analyses of cohorts representing the general population for stroke.**

| Marker | Outcome | Risk Applies To | Risk | Results | 95% ci | N Patients | N Cohorts | Het. | Pooling Method | Adj. | Patient group | IPD | Follow-up Period | Publication |
| --- | --- | --- | --- | --- | --- | --- | --- | --- | --- | --- | --- | --- | --- | --- |
|  |  |  |  |  |  |  |  |  |  |  |  |  |  |  |
| ***Inflammation*** |  |  |  |  |  |  |  |  |  |  |  |  |  |  |
| CRP | Stroke | Top vs bottom tertile | RR | 1.68 | 1.40 - 2.01 | 9,572 total | 4 | no | Fixed effects model | yes | General population | no | > 8 yr | [82] |
|  |  |  |  |  |  |  |  |  |  |  |  |  |  |  |
| ***Lipids*** |  |  |  |  |  |  |  |  |  |  |  |  |  |  |
| Triglycerides | Ischemic stroke | 1 SD increase | HR | 1.35 | 1.00 - 1.83 | 273 cases | 13 | no | Cox regression | yes | Asia-Pacific population | yes | 2.5 - 19.7 yr | [58] |
| Cholesterol | Ischemic stroke | Increase: <4.2 , 4.2-4.6, 4.7-5.1, 5.2-5.8, >5.8 mmol/L | HR | 1.15 | 0.99 - 1.34 | 420 cases | 24 | no | Cox regression | yes | Asia-Pacific population | yes | 2.7 - 25.1 yr | [59] |
| Triglycerides | Ischemic stroke | Increase: <0.8, 0.8-1.0, 1.1-1.3, 1.4-1.8, >1.8 mmol/L | HR | 1.03 | 0.74 - 1.45 | 420 cases | 24 | no | Cox regression | yes | Asia-Pacific population | yes | 2.7 - 25.1 yr | [59] |

**Table S9. Meta-analyses of cohorts with and without pre-existing cardiovascular disease for stroke.**

| Marker | Outcome | Risk Applies To | Risk | Results | 95% ci | N Patients | N Cohorts | Het. | Pooling Method | Adj. | Patient group, general population | IPD | Follow-up Period | Publication |
| --- | --- | --- | --- | --- | --- | --- | --- | --- | --- | --- | --- | --- | --- | --- |
|  |  |  |  |  |  |  |  |  |  |  |  |  |  |  |
| ***Ischemia*** |  |  |  |  |  |  |  |  |  |  |  |  |  |  |
| BNP + NT-proBNP | Stroke | Top vs bottom tertile | RR | 1.93 | 1.58 - 2.37 | 2,063 cases | 13 | yes | Random effects model | yes | Population based, high CVD risk, pre-existing CVD | no | 5 yr mean | [71] |
|  |  |  |  |  |  |  |  |  |  |  |  |  |  |  |
| ***Kidney function*** |  |  |  |  |  |  |  |  |  |  |  |  |  |  |
| Cystatin C | Stroke | Top vs bottom quintile | RR | 1.83 | 1.12 - 3.00 | 828 cases | 4 | yes | Random effects model | yes | Population based, elderly, CVD patients | no | 3.1 - 7.4 yr | [45] |
|  |  |  |  |  |  |  |  |  |  |  |  |  |  |  |
| ***Lipids*** |  |  |  |  |  |  |  |  |  |  |  |  |  |  |
| Cholesterol | Ischemic stroke | Top vs bottom quintile | HR | 1.5 | 1.3 - 1.8 | 749 cases | 15 | yes | Cox regression | yes | Not selected on any disease, Asia Pacific region | yes | 2.7 - 24.6 yr | [61] |
| Lipoprotein(a) | Stroke | Top vs tertile | RR | 1.21 | 1.04 - 1.41 | 2,009 cases | 8 | no | Random effects model | yes | Stroke and non-stroke patients | no | 3 - 14 yr | [83] |
| Triglycerides | Ischemic stroke, incl. TIA | 1 SD increase | RR | 1.11 | 1.08 - 1.15 | 2,046 cases | 5 | no | Inverse variance weighted mean | yes | Population based, CHD patients free of stroke | no | 3 - 18 yr | [84] |
| Lp-PLA2 | Ischemic stroke | 1 SD increase | RR | 1.10 | 1.04 - 1.16 | 2,097 cases | 5 | no | Fixed effects model | yes | No pre-existing CVD, stable CHD | no | 5.8 yr median | [47] |
| Triglycerides | Stroke | 10 mg/dL increase | RR | 1.05 | 1.03 - 1.07 | 3,348 cases | 29 | n.a. | Regression | yes | Primary and secondary events | no | >1yr | [85] |
|  |  |  |  |  |  |  |  |  |  |  |  |  |  |  |
| ***Miscellaneous*** |  |  |  |  |  |  |  |  |  |  |  |  |  |  |
| Homocysteine | Stroke | 5 μmol/L increase | OR | 1.42 | 1.21 - 1.66 | 676 cases | 8 | n.a. | Random effects model | yes | No renal disease | no | 3 - 12 yr | [77] |

References

1. Coutinho M, Gerstein HC, Wang Y, Yusuf S (1999) The relationship between glucose and incident cardiovascular events. A metaregression analysis of published data from 20 studies of 95,783 individuals followed for 12.4 years. Diabetes Care 22: 233-240.

2. Santos-Oliveira R, Purdy C, da Silva MP, dos Anjos Carneiro-Leao AM, Machado M, et al. (2011) Haemoglobin A1c levels and subsequent cardiovascular disease in persons without diabetes: a meta-analysis of prospective cohorts. Diabetologia 54: 1327-1334.

3. Sarwar N, Aspelund T, Eiriksdottir G, Gobin R, Seshasai SR, et al. (2010) Markers of dysglycaemia and risk of coronary heart disease in people without diabetes: Reykjavik prospective study and systematic review. PLoS Med 7: e1000278.

4. Danesh J, Lewington S, Thompson SG, Lowe GD, Collins R, et al. (2005) Plasma fibrinogen level and the risk of major cardiovascular diseases and nonvascular mortality: an individual participant meta-analysis. Jama 294: 1799-1809.

5. Danesh J, Wheeler JG, Hirschfield GM, Eda S, Eiriksdottir G, et al. (2004) C-reactive protein and other circulating markers of inflammation in the prediction of coronary heart disease. N Engl J Med 350: 1387-1397.

6. Grandi NC, Breitling LP, Brenner H (2010) Vitamin D and cardiovascular disease: systematic review and meta-analysis of prospective studies. Prev Med 51: 228-233.

7. Singh S, Duggal J, Molnar J, Maldonado F, Barsano CP, et al. (2008) Impact of subclinical thyroid disorders on coronary heart disease, cardiovascular and all-cause mortality: a meta-analysis. Int J Cardiol 125: 41-48.

8. Ruige JB, Mahmoud AM, De Bacquer D, Kaufman JM (2011) Endogenous testosterone and cardiovascular disease in healthy men: a meta-analysis. Heart 97: 870-875.

9. Shah T, Casas JP, Cooper JA, Tzoulaki I, Sofat R, et al. (2009) Critical appraisal of CRP measurement for the prediction of coronary heart disease events: new data and systematic review of 31 prospective cohorts. Int J Epidemiol 38: 217-231.

10. Kaptoge S, Di Angelantonio E, Lowe G, Pepys MB, Thompson SG, et al. (2010) C-reactive protein concentration and risk of coronary heart disease, stroke, and mortality: an individual participant meta-analysis. Lancet 375: 132-140.

11. Danesh J, Kaptoge S, Mann AG, Sarwar N, Wood A, et al. (2008) Long-term interleukin-6 levels and subsequent risk of coronary heart disease: two new prospective studies and a systematic review. PLoS Med 5: e78.

12. Di Angelantonio E, Danesh J, Eiriksdottir G, Gudnason V (2007) Renal function and risk of coronary heart disease in general populations: new prospective study and systematic review. PLoS Med 4: e270.

13. Kim SY, Guevara JP, Kim KM, Choi HK, Heitjan DF, et al. (2010) Hyperuricemia and coronary heart disease: a systematic review and meta-analysis. Arthritis Care Res (Hoboken) 62: 170-180.

14. Thompson A, Danesh J (2006) Associations between apolipoprotein B, apolipoprotein AI, the apolipoprotein B/AI ratio and coronary heart disease: a literature-based meta-analysis of prospective studies. J Intern Med 259: 481-492.

15. Lewington S, Whitlock G, Clarke R, Sherliker P, Emberson J, et al. (2007) Blood cholesterol and vascular mortality by age, sex, and blood pressure: a meta-analysis of individual data from 61 prospective studies with 55,000 vascular deaths. Lancet 370: 1829-1839.

16. Sarwar N, Danesh J, Eiriksdottir G, Sigurdsson G, Wareham N, et al. (2007) Triglycerides and the risk of coronary heart disease: 10,158 incident cases among 262,525 participants in 29 Western prospective studies. Circulation 115: 450-458.

17. Di Angelantonio E, Sarwar N, Perry P, Kaptoge S, Ray KK, et al. (2009) Major lipids, apolipoproteins, and risk of vascular disease. Jama 302: 1993-2000.

18. Nakagami T, Qiao Q, Tuomilehto J, Balkau B, Tajima N, et al. (2006) Screen-detected diabetes, hypertension and hypercholesterolemia as predictors of cardiovascular mortality in five populations of Asian origin: the DECODA study. Eur J Cardiovasc Prev Rehabil 13: 555-561.

19. Anum EA, Adera T (2004) Hypercholesterolemia and coronary heart disease in the elderly: a meta-analysis. Ann Epidemiol 14: 705-721.

20. Erqou S, Kaptoge S, Perry PL, Di Angelantonio E, Thompson A, et al. (2009) Lipoprotein(a) concentration and the risk of coronary heart disease, stroke, and nonvascular mortality. Jama 302: 412-423.

21. Bautista LE, Arenas IA, Penuela A, Martinez LX (2002) Total plasma homocysteine level and risk of cardiovascular disease: a meta-analysis of prospective cohort studies. J Clin Epidemiol 55: 882-887.

22. Ford ES, Smith SJ, Stroup DF, Steinberg KK, Mueller PW, et al. (2002) Homocyst(e)ine and cardiovascular disease: a systematic review of the evidence with special emphasis on case-control studies and nested case-control studies. Int J Epidemiol 31: 59-70.

23. Humphrey LL, Fu R, Rogers K, Freeman M, Helfand M (2008) Homocysteine level and coronary heart disease incidence: a systematic review and meta-analysis. Mayo Clin Proc 83: 1203-1212.

24. (2002) Homocysteine and risk of ischemic heart disease and stroke: a meta-analysis. Jama 288: 2015-2022.

25. Flores-Mateo G, Navas-Acien A, Pastor-Barriuso R, Guallar E (2006) Selenium and coronary heart disease: a meta-analysis. Am J Clin Nutr 84: 762-773.

26. Whincup PH, Danesh J, Walker M, Lennon L, Thomson A, et al. (2002) von Willebrand factor and coronary heart disease: prospective study and meta-analysis. Eur Heart J 23: 1764-1770.

27. Rothwell PM, Howard SC, Power DA, Gutnikov SA, Algra A, et al. (2004) Fibrinogen concentration and risk of ischemic stroke and acute coronary events in 5113 patients with transient ischemic attack and minor ischemic stroke. Stroke 35: 2300-2305.

28. Lowe GD, Danesh J, Lewington S, Walker M, Lennon L, et al. (2004) Tissue plasminogen activator antigen and coronary heart disease. Prospective study and meta-analysis. Eur Heart J 25: 252-259.

29. Padayachee L, Rodseth RN, Biccard BM (2009) A meta-analysis of the utility of C-reactive protein in predicting early, intermediate-term and long term mortality and major adverse cardiac events in vascular surgical patients. Anaesthesia 64: 416-424.

30. Hemingway H, Philipson P, Chen R, Fitzpatrick NK, Damant J, et al. (2010) Evaluating the quality of research into a single prognostic biomarker: a systematic review and meta-analysis of 83 studies of C-reactive protein in stable coronary artery disease. PLoS Med 7: e1000286.

31. Danesh J, Whincup P, Walker M, Lennon L, Thomson A, et al. (2000) Low grade inflammation and coronary heart disease: prospective study and updated meta-analyses. Bmj 321: 199-204.

32. Whiteley W, Jackson C, Lewis S, Lowe G, Rumley A, et al. (2009) Inflammatory markers and poor outcome after stroke: a prospective cohort study and systematic review of interleukin-6. PLoS Med 6: e1000145.

33. Gurm HS, Bhatt DL, Lincoff AM, Tcheng JE, Kereiakes DJ, et al. (2003) Impact of preprocedural white blood cell count on long term mortality after percutaneous coronary intervention: insights from the EPIC, EPILOG, and EPISTENT trials. Heart 89: 1200-1204.

34. Ottani F, Galvani M, Nicolini FA, Ferrini D, Pozzati A, et al. (2000) Elevated cardiac troponin levels predict the risk of adverse outcome in patients with acute coronary syndromes. Am Heart J 140: 917-927.

35. Rodseth RN, Lurati Buse GA, Bolliger D, Burkhart CS, Cuthbertson BH, et al. (2011) The predictive ability of pre-operative B-type natriuretic peptide in vascular patients for major adverse cardiac events: an individual patient data meta-analysis. J Am Coll Cardiol 58: 522-529.

36. Heidenreich PA, Go A, Melsop KA, Alloggiamento T, McDonald KM, et al. (2000) Prediction of risk for patients with unstable angina. Evid Rep Technol Assess (Summ): 1-3.

37. Fleming SM, Daly KM (2001) Cardiac troponins in suspected acute coronary syndrome: a meta-analysis of published trials. Cardiology 95: 66-73.

38. Wu AH, Lane PL (1995) Metaanalysis in clinical chemistry: validation of cardiac troponin T as a marker for ischemic heart diseases. Clin Chem 41: 1228-1233.

39. Olatidoye AG, Wu AH, Feng YJ, Waters D (1998) Prognostic role of troponin T versus troponin I in unstable angina pectoris for cardiac events with meta-analysis comparing published studies. Am J Cardiol 81: 1405-1410.

40. Heidenreich PA, Alloggiamento T, Melsop K, McDonald KM, Go AS, et al. (2001) The prognostic value of troponin in patients with non-ST elevation acute coronary syndromes: a meta-analysis. J Am Coll Cardiol 38: 478-485.

41. Wu AH, Boden WE, McKay RG (2002) Long-term follow-up of patients with increased cardiac troponin concentrations following percutaneous coronary intervention. Am J Cardiol 89: 1300-1302.

42. Feldman DN, Kim L, Rene AG, Minutello RM, Bergman G, et al. (2011) Prognostic value of cardiac troponin-I or troponin-T elevation following nonemergent percutaneous coronary intervention: a meta-analysis. Catheter Cardiovasc Interv 77: 1020-1030.

43. Nienhuis MB, Ottervanger JP, Bilo HJ, Dikkeschei BD, Zijlstra F (2008) Prognostic value of troponin after elective percutaneous coronary intervention: A meta-analysis. Catheter Cardiovasc Interv 71: 318-324.

44. van der Velde M, Matsushita K, Coresh J, Astor BC, Woodward M, et al. (2011) Lower estimated glomerular filtration rate and higher albuminuria are associated with all-cause and cardiovascular mortality. A collaborative meta-analysis of high-risk population cohorts. Kidney Int 79: 1341-1352.

45. Lee M, Saver JL, Huang WH, Chow J, Chang KH, et al. (2010) Impact of elevated cystatin C level on cardiovascular disease risk in predominantly high cardiovascular risk populations: a meta-analysis. Circ Cardiovasc Qual Outcomes 3: 675-683.

46. Danesh J, Collins R, Peto R (2000) Lipoprotein(a) and coronary heart disease. Meta-analysis of prospective studies. Circulation 102: 1082-1085.

47. Thompson A, Gao P, Orfei L, Watson S, Di Angelantonio E, et al. (2010) Lipoprotein-associated phospholipase A(2) and risk of coronary disease, stroke, and mortality: collaborative analysis of 32 prospective studies. Lancet 375: 1536-1544.

48. Danesh J, Collins R, Peto R, Lowe GD (2000) Haematocrit, viscosity, erythrocyte sedimentation rate: meta-analyses of prospective studies of coronary heart disease. Eur Heart J 21: 515-520.

49. Kim SY, Guevara JP, Kim KM, Choi HK, Heitjan DF, et al. (2009) Hyperuricemia and risk of stroke: a systematic review and meta-analysis. Arthritis Rheum 61: 885-892.

50. Hu G, Qiao Q, Tuomilehto J, Eliasson M, Feskens EJ, et al. (2004) Plasma insulin and cardiovascular mortality in non-diabetic European men and women: a meta-analysis of data from eleven prospective studies. Diabetologia 47: 1245-1256.

51. Sarwar N, Sattar N, Gudnason V, Danesh J (2007) Circulating concentrations of insulin markers and coronary heart disease: a quantitative review of 19 Western prospective studies. Eur Heart J 28: 2491-2497.

52. Ruige JB, Assendelft WJ, Dekker JM, Kostense PJ, Heine RJ, et al. (1998) Insulin and risk of cardiovascular disease: a meta-analysis. Circulation 97: 996-1001.

53. Maresca G, Di Blasio A, Marchioli R, Di Minno G (1999) Measuring plasma fibrinogen to predict stroke and myocardial infarction: an update. Arterioscler Thromb Vasc Biol 19: 1368-1377.

54. Ernst E, Resch KL (1993) Fibrinogen as a cardiovascular risk factor: a meta-analysis and review of the literature. Ann Intern Med 118: 956-963.

55. Razvi S, Shakoor A, Vanderpump M, Weaver JU, Pearce SH (2008) The influence of age on the relationship between subclinical hypothyroidism and ischemic heart disease: a metaanalysis. J Clin Endocrinol Metab 93: 2998-3007.

56. Ochs N, Auer R, Bauer DC, Nanchen D, Gussekloo J, et al. (2008) Meta-analysis: subclinical thyroid dysfunction and the risk for coronary heart disease and mortality. Ann Intern Med 148: 832-845.

57. Danesh J, Collins R, Appleby P, Peto R (1998) Association of fibrinogen, C-reactive protein, albumin, or leukocyte count with coronary heart disease: meta-analyses of prospective studies. Jama 279: 1477-1482.

58. Patel A, Barzi F, Jamrozik K, Lam TH, Ueshima H, et al. (2004) Serum triglycerides as a risk factor for cardiovascular diseases in the Asia-Pacific region. Circulation 110: 2678-2686.

59. Woodward M, Huxley H, Lam TH, Barzi F, Lawes CM, et al. (2005) A comparison of the associations between risk factors and cardiovascular disease in Asia and Australasia. Eur J Cardiovasc Prev Rehabil 12: 484-491.

60. Austin MA, Hokanson JE, Edwards KL (1998) Hypertriglyceridemia as a cardiovascular risk factor. Am J Cardiol 81: 7B-12B.

61. Zhang X, Patel A, Horibe H, Wu Z, Barzi F, et al. (2003) Cholesterol, coronary heart disease, and stroke in the Asia Pacific region. Int J Epidemiol 32: 563-572.

62. Levitan EB, Song Y, Ford ES, Liu S (2004) Is nondiabetic hyperglycemia a risk factor for cardiovascular disease? A meta-analysis of prospective studies. Arch Intern Med 164: 2147-2155.

63. Danesh J, Whincup P, Walker M, Lennon L, Thomson A, et al. (2001) Fibrin D-dimer and coronary heart disease: prospective study and meta-analysis. Circulation 103: 2323-2327.

64. Sattar N, Wannamethee G, Sarwar N, Tchernova J, Cherry L, et al. (2006) Adiponectin and coronary heart disease: a prospective study and meta-analysis. Circulation 114: 623-629.

65. Danesh J, Whincup P, Lewington S, Walker M, Lennon L, et al. (2002) Chlamydia pneumoniae IgA titres and coronary heart disease; prospective study and meta-analysis. Eur Heart J 23: 371-375.

66. Danesh J, Whincup P, Walker M, Lennon L, Thomson A, et al. (2000) Chlamydia pneumoniae IgG titres and coronary heart disease: prospective study and meta-analysis. Bmj 321: 208-213.

67. Buckley DI, Fu R, Freeman M, Rogers K, Helfand M (2009) C-reactive protein as a risk factor for coronary heart disease: a systematic review and meta-analyses for the U.S. Preventive Services Task Force. Ann Intern Med 151: 483-495.

68. Wheeler JG, Mussolino ME, Gillum RF, Danesh J (2004) Associations between differential leucocyte count and incident coronary heart disease: 1764 incident cases from seven prospective studies of 30,374 individuals. Eur Heart J 25: 1287-1292.

69. Malik I, Danesh J, Whincup P, Bhatia V, Papacosta O, et al. (2001) Soluble adhesion molecules and prediction of coronary heart disease: a prospective study and meta-analysis. Lancet 358: 971-976.

70. Jefferis BJ, Papacosta O, Owen CG, Wannamethee SG, Humphries SE, et al. (2011) Interleukin 18 and coronary heart disease: prospective study and systematic review. Atherosclerosis 217: 227-233.

71. Di Angelantonio E, Chowdhury R, Sarwar N, Ray KK, Gobin R, et al. (2009) B-type natriuretic peptides and cardiovascular risk: systematic review and meta-analysis of 40 prospective studies. Circulation 120: 2177-2187.

72. Wheeler JG, Juzwishin KD, Eiriksdottir G, Gudnason V, Danesh J (2005) Serum uric acid and coronary heart disease in 9,458 incident cases and 155,084 controls: prospective study and meta-analysis. PLoS Med 2: e76.

73. Garza CA, Montori VM, McConnell JP, Somers VK, Kullo IJ, et al. (2007) Association between lipoprotein-associated phospholipase A2 and cardiovascular disease: a systematic review. Mayo Clin Proc 82: 159-165.

74. Sniderman AD, Williams K, Contois JH, Monroe HM, McQueen MJ, et al. (2011) A meta-analysis of low-density lipoprotein cholesterol, non-high-density lipoprotein cholesterol, and apolipoprotein B as markers of cardiovascular risk. Circ Cardiovasc Qual Outcomes 4: 337-345.

75. Moller J, Nielsen GM, Tvedegaard KC, Andersen NT, Jorgensen PE (2000) A meta-analysis of cerebrovascular disease and hyperhomocysteinaemia. Scand J Clin Lab Invest 60: 491-499.

76. Danesh J, Lewington S (1998) Plasma homocysteine and coronary heart disease: systematic review of published epidemiological studies. J Cardiovasc Risk 5: 229-232.

77. Wald DS, Law M, Morris JK (2002) Homocysteine and cardiovascular disease: evidence on causality from a meta-analysis. Bmj 325: 1202.

78. Danesh J, Appleby P (1999) Coronary heart disease and iron status: meta-analyses of prospective studies. Circulation 99: 852-854.

79. Rodseth RN, Padayachee L, Biccard BM (2008) A meta-analysis of the utility of pre-operative brain natriuretic peptide in predicting early and intermediate-term mortality and major adverse cardiac events in vascular surgical patients. Anaesthesia 63: 1226-1233.

80. Ryding AD, Kumar S, Worthington AM, Burgess D (2009) Prognostic value of brain natriuretic peptide in noncardiac surgery: a meta-analysis. Anesthesiology 111: 311-319.

81. Gueyffier F, Boissel JP, Pocock S, Boutitie F, Coope J, et al. (1999) Identification of risk factors in hypertensive patients: contribution of randomized controlled trials through an individual patient database. Circulation 100: e88-94.

82. Kuo HK, Yen CJ, Chang CH, Kuo CK, Chen JH, et al. (2005) Relation of C-reactive protein to stroke, cognitive disorders, and depression in the general population: systematic review and meta-analysis. Lancet Neurol 4: 371-380.

83. Smolders B, Lemmens R, Thijs V (2007) Lipoprotein (a) and stroke: a meta-analysis of observational studies. Stroke 38: 1959-1966.

84. Labreuche J, Touboul PJ, Amarenco P (2009) Plasma triglyceride levels and risk of stroke and carotid atherosclerosis: a systematic review of the epidemiological studies. Atherosclerosis 203: 331-345.

85. Labreuche J, Deplanque D, Touboul PJ, Bruckert E, Amarenco P (2010) Association between change in plasma triglyceride levels and risk of stroke and carotid atherosclerosis: systematic review and meta-regression analysis. Atherosclerosis 212: 9-15.

1. 95% ci: 95% confidence interval [↑](#footnote-ref-2)
2. Het.: heterogeneity between cohorts. [↑](#footnote-ref-3)
3. Adj.: adjustment performed with other risk factors. [↑](#footnote-ref-4)
4. IPD: individual patient data. [↑](#footnote-ref-5)
5. CVD: cardiovascular disease [↑](#footnote-ref-6)
6. n.a.: not available. [↑](#footnote-ref-7)
7. CHD: coronary heart disease [↑](#footnote-ref-8)
8. MI: myocardial infarction [↑](#footnote-ref-9)
9. SD: standard deviation [↑](#footnote-ref-10)
10. IHD: ischemic heart disease [↑](#footnote-ref-11)
11. CRP: C-reactive protein [↑](#footnote-ref-12)
12. eGFR: estimated glomerular filtration rate. [↑](#footnote-ref-13)
13. Apo: apolipoprotein [↑](#footnote-ref-14)
14. HDL: high density lipoprotein [↑](#footnote-ref-15)
15. hs: high sensitivity [↑](#footnote-ref-16)
16. MACE: major adverse cardiac events. [↑](#footnote-ref-17)
17. PCI: percutaneous coronary intervention. [↑](#footnote-ref-18)
18. cTn: cardiac troponin [↑](#footnote-ref-19)
19. (NT-pro)BNP: (N-terminal prohormone of) brain natriuretic peptide. [↑](#footnote-ref-20)
20. ACS: acute coronary syndrome. [↑](#footnote-ref-21)
21. (n)STEMI: (non)-ST elevated myocardial infarction. [↑](#footnote-ref-22)
22. AMI: acute myocardial infarction. [↑](#footnote-ref-23)
23. CKD: chronic kidney disease. [↑](#footnote-ref-24)
24. Lp-PLA2: lipoprotein associated phospholipase A2. [↑](#footnote-ref-25)
25. TIA: transient ischemic attack. [↑](#footnote-ref-26)
26. HF: heart failure. [↑](#footnote-ref-27)
27. ICAM: intercellular adhesion molecule [↑](#footnote-ref-28)
28. CAD: coronary artery disease. [↑](#footnote-ref-29)
29. VCAM: vascular cell adhesion molecule. [↑](#footnote-ref-30)
30. LDL: low density lipoprotein. [↑](#footnote-ref-31)
